# TSPYL1 as a Critical Regulator of TGFβ Signaling through Repression of TGFBR1 and TSPYL2

**DOI:** 10.1002/advs.202306486

**Published:** 2024-04-08

**Authors:** Huiqi Tan, Mia Xinfang Miao, Rylee Xu Luo, Joan So, Lei Peng, Xiaoxuan Zhu, Eva Hin Wa Leung, Lina Zhu, Kui Ming Chan, Martin Cheung, Siu Yuen Chan

**Affiliations:** ^1^ Department of Paediatrics and Adolescent Medicine School of Clinical Medicine Li Ka Shing Faculty of Medicine The University of Hong Kong Hong Kong China; ^2^ Department of Biomedical Sciences The City University of Hong Kong Hong Kong China; ^3^ School of Biomedical Sciences Li Ka Shing Faculty of Medicine The University of Hong Kong Hong Kong China

**Keywords:** FOXA1, nucleosome assembly protein, TGFBR1, TGFβ signaling, TSPYL1, TSPYL2

## Abstract

Nucleosome assembly proteins (NAPs) have been identified as histone chaperons. Testis‐Specific Protein, Y‐Encoded‐Like (TSPYL) is a newly arisen NAP family in mammals. TSPYL2 can be transcriptionally induced by DNA damage and TGFβ causing proliferation arrest. TSPYL1, another TSPYL family member, has been poorly characterized and is the only TSPYL family member known to be causal of a lethal recessive disease in humans. This study shows that TSPYL1 and TSPYL2 play an opposite role in TGFβ signaling. TSPYL1 partners with the transcription factor FOXA1 and histone methyltransferase EZH2, and at the same time represses *TGFBR1* and epithelial‐mesenchymal transition (EMT). Depletion of TSPYL1 increases *TGFBR1* expression, upregulates TGFβ signaling, and elevates the protein stability of TSPYL2. Intriguingly, TSPYL2 forms part of the SMAD2/3/4 signal transduction complex upon stimulation by TGFβ to execute the transcriptional responses. Depletion of TSPYL2 rescues the EMT phenotype of TSPYL1 knockdown in A549 lung carcinoma cells. The data demonstrates the prime role of TSPYL2 in causing the dramatic defects in TSPYL1 deficiency. An intricate counter‐balancing role of TSPYL1 and TSPYL2 in regulating TGFβ signaling is also unraveled.

## Graphical abstract

TSPYL1 partners with FOXA1 and EZH2 to repress *TGFBR1* transcription, thereby mitigating the level of TGFβ receptors and sensitivity to TGFβ. TSPYL1 deficiency enhances the response to TGFβ and stabilizes the related protein TSPYL2. TSPYL2 interacts with the SMAD2/3/4 complex to regulate transcription and hence executes the context‐dependent TGFβ driven events including epithelial mesenchymal transition in BE(2)‐C and A549 cells.

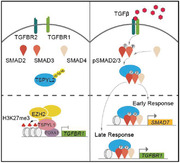

## 1 Introduction

Transforming growth factor β (TGFβ) signaling can regulate cell growth, epithelial‐mesenchymal transition (EMT), differentiation, and homeostasis.^[^ ^1^ ^]^ The receptor for TGFβ peptide is a heteromeric complex of TGFBR1 (also called ALK5) and TGFΒR2, which phosphorylates SMAD2 and SMAD3 to form a complex with SMAD4 upon ligand binding. The phospho‐SMAD complex is responsible for executing the early transcription response in TGFβ signaling. SMAD3 and SMAD4 recognize specific DNA sequences to determine gene expression.^[^ ^2^ ^]^ However, the DNA binding affinity of SMAD3 and SMAD4 is relatively weak, so interaction of the SMAD complex with other context‐specific transcription factors is required for transcription regulation.^[^ ^2^, ^3^ ^]^ The control of TGFβ signaling in vivo is complex. Besides the bioavailability of active TGFβ, the low abundance of cell surface receptors and a very high ligand binding affinity allow the cell to exquisitely regulate its ligand sensitivity. For example, regulated expression of TGFBR1 determines T cell quiescence and activation,^[^ ^4^ ^]^ and late‐stage adult neurogenesis.^[^ ^5^ ^]^ Besides playing a central role in development and homeostasis, TGFβ also participates in disease processes such as cancer and organ fibrosis.

TSPYL2 (also named CDA1, CINAP, DENTT, and NP79) belongs to the Nucleosome Assembly Protein (NAP) superfamily. NAPs are histone chaperones for the specific association of DNA and histones, and they also regulate transcription through facilitating the dynamic binding of protein complexes to chromatin.^[^ ^6^ ^]^ Previous data show that overexpression (OE) of TSPYL2 augments the phosphorylation of SMAD2,^[^ ^7^ ^]^ and SMAD3 upon the addition of TGFβ.^[^ ^8^ ^]^ Furthermore, proliferation arrest induced by overexpressing TSPYL2 is dependent on SMAD4 in A549 lung carcinoma cells; whereas knockdown (KD) of TSPYL2 confers resistance to TGFβ induced proliferation arrest in HaCaT human keratinocytes.^[^ ^7^ ^]^ Pointing toward the in vivo significance of TSPYL2 in TGFβ signaling, TSPYL2 protein increases upon stimulation by TGFβ in mouse vascular smooth muscle cells,^[^ ^8a^ ^]^ and targeting TSPYL2 specifically retards renal extracellular matrix accumulation and fibrosis in a diabetic mouse model.^[^ ^9^ ^]^ However, how TSPYL2 participates in TGFβ signaling is yet to be established.

There are 6 TSPYL genes in humans, namely TSPYL1 to TSPYL6, with TSPYL3 being a pseudogene. TSPYL1, 2, 4, and 5 have ubiquitous expression at variable expression levels, while TSPYL6 is expressed exclusively in the testes from GTEX datasets (https://www.gtexportal.org/). TSPYL1,2, 4, and 5 regulate the expression of specific CYP genes.^[^ ^10^ ^]^ Besides, TSPYL2 regulates the expression of neuronal genes.^[^ ^11^ ^]^ Importantly, TSPYL1 is essential for survival. Loss‐of‐function mutations of *TSPYL1* cause sudden infant death with dysgenesis of testes syndrome (SIDDT).^[^ ^12^ ^]^ Patients presented with multiple signs of visceroautonomic dysfunction and died before one‐year‐old from cardiac or respiratory arrest. *Tspyl1* knockout (KO) mice also have retarded growth and most die around the weaning stage, but there is no testis dysgenesis.^[^ ^13^ ^]^ Despite the vital importance of TSPYL1, little is known about its function.

When we examined the importance of TSPYL1 in neural proliferation and differentiation in a neuroblastoma cell line BE(2)‐C, intriguingly KO of *TSPYL1* caused a phenotype consistent with EMT. Here we establish that TSPYL1 depletion drives EMT. Mechanistically, TSPYL1 interacts with the transcription factor FOXA1 and histone methyltransferase EZH2 to repress the transcription of *TGFBR1*. The augmented TGFβ signaling upon *TSPYL1* depletion stabilizes TSPYL2, which interacts with the SMAD2/3/4 complex to activate TGFβ target genes including *TGFBR1*, and mediates the EMT phenotype. Our data provide insight into the biological significance of TSPYL1 in controlling TGFβ signaling and in counterbalancing the effects of TSPYL2.

## 2 Results

### 2.1 TSPYL1 Depletion Drives EMT through TGFβ Signaling

**Figure 1:**
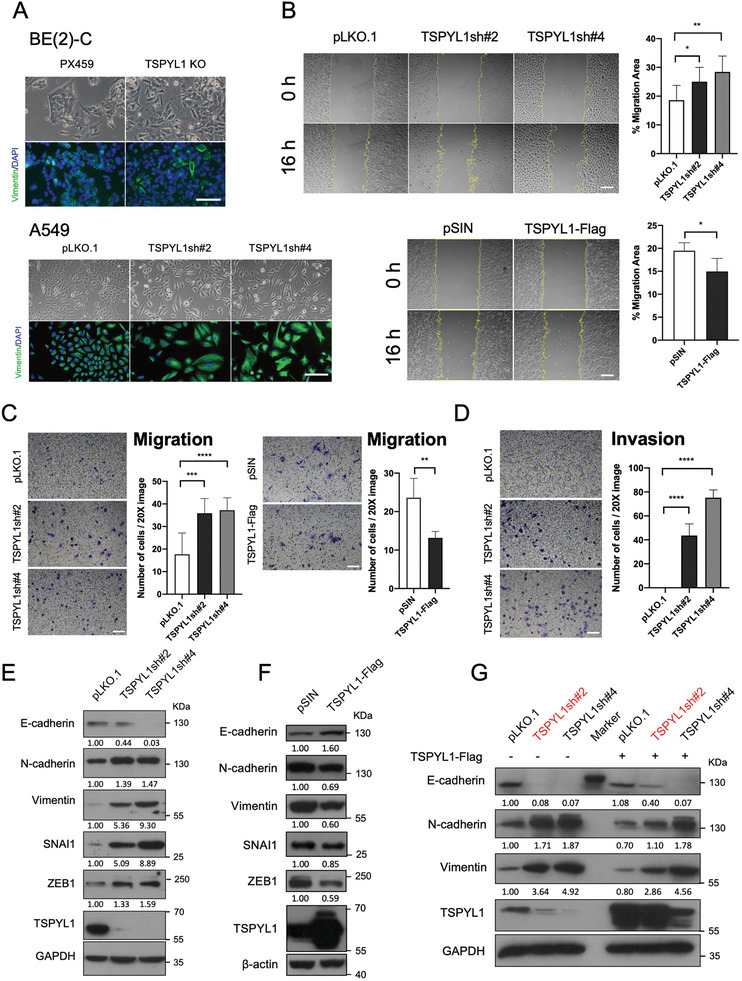
TSPYL1 depletion drives EMT. A) Representative images of control, TSPYL1 knockout (KO) BE(2)‐C clones and knockdown A549 cells. TSPYL1 KO BE(2)‐C clones were generated by transfection with sgRNA cloned into CRISPR/Cas9 vector PX459. Transfected cells were selected with puromycin and single colonies were picked. An image of one representative clone was shown. A549 cells were transduced with empty vector pLKO.1 or two different TSPYL1sh for 48 h, and images were taken 7 days post‐transduction (i.e. removal of viruses). Scale bar: 100 µm. B) Confluent TSPYL1 knocked down or overexpression A549 cells were treated with Mitomycin C for 2 h before wounding. Images were taken immediately (0 h) and 16 h afterward. Cell borders were outlined. The percentage of migrated area was measured by Image J and presented on the right. Data are shown as the mean ± S.D. *n* ≥ 4 wells, ^*^ *p* < 0.05, ^**^ *p* < 0.01 by unpaired Student's *t*‐test. Scale bar: 100 µm. C, D) A549 cells were seeded onto Transwell directly (C) or with precoating of Matrigel (D). Transwells were removed 24 h later. Cells that had migrated through the transwell were stained with crystal violet. Images were taken under 20X magnification and the cell number was counted. Data are shown as the mean ± S.D. *n* ≥ 5 images, ^**^ *p* < 0.01, ^***^ *p* < 0.001, ^****^ *p* < 0.0001 by unpaired Student's *t*‐test. Scale bar: 100 µm. E) The protein levels of epithelial marker E‐cadherin and mesenchymal markers as indicated were determined by immunoblotting in control or knockdown A549 cells on day 9 after transduction. F) The protein levels of epithelial marker E‐cadherin and mesenchymal markers as indicated were determined by immunoblotting in A549 cells transduced with empty vector pSIN or TSPYL1‐Flag 3 days post‐transduction. G) Control or TSPYL1 knockdown A549 cells were transduced with empty vector or TSPYL1‐Flag. Protein samples were collected after 72 h for immunoblotting of proteins as indicated. The highlighted pair showed efficient rescue by TSPYL1‐Flag with TSPYL1sh#2, targeting the endogenous but not ectopic *TSPYL1* cDNA.

Since TSPYL1 is critical for the normal functioning of the central nervous system as indicated in SIDDT, we explored whether KO of *TSPYL1* in the neuroblastoma cell line BE(2)‐C affected differentiation in vitro. KO of *TSPYL1* in BE(2)‐C cells resulted in enlarged cells (**Figure** **1**A). There were enhanced migratory and invasive abilities, together with increased transcript levels of *SNAI1* and *SNAI2* encoding the EMT transcription factors SNAI1 and SLUG (Figure S1A–D, Supporting Information). These changes are typical during neural crest cell migration and differentiation.^[^ ^14^ ^]^ To establish that the loss of TSPYL1 can trigger EMT in epithelial cells, we employed epithelial cell lines including lung carcinoma A549 and breast ductal carcinoma MCF7. We knocked down TSPYL1 by lentiviral constructs carrying two independent short hairpin RNAs (shRNAs). TSPYL1sh#2 targets the 3’UTR of *TSPYL1* while TSPYL1sh#4 targets the coding region. TSPYL1 KD in A549 and MCF7 cells adversely affected their proliferation (Figure S2, Supporting Information) and in A549, fibroblast‐like morphological changes were also observed (Figure 1A). Therefore, we performed a mechanistic study for EMT in A549 cells. First, we conducted wound healing and transwell assays to ascertain EMT behavior. Compared to the control group transduced with the transfer vector pLKO.1, TSPYL1 KD cells migrated significantly faster across the wound or the transwell, while TSPYL1 OE had the opposite effect (Figure 1B,C). Invasion assay using Matrigel‐coated transwells further indicated that TSPYL1 KD triggered cell invasion (Figure 1D). These behavioral changes, accompanied by reduced cell proliferation, did not increase the metastatic potential when the TSPYL1‐depleted A549 cells were injected into nude mice (Figure S3, Supporting Information). At the molecular level, TSPYL1 KD resulted in reduced E‐cadherin and increased mesenchymal marker levels including N‐cadherin, Vimentin, SNAI1, and ZEB1 (Figure 1E). Conversely, OE of TSPYL1 increased E‐cadherin and decreased N‐cadherin, Vimentin, SNAI1, and ZEB1 levels (Figure 1F). In the genetic rescue, re‐introduction of TSPYL1 in TSPYL1 KD A549 cells increased the expression of E‐cadherin and decreased the expression of N‐cadherin and Vimentin after 3 days. As highlighted, the rescue was more efficient for TSPYL1sh#2 than TSPYL1sh#4 KD cells (Figure 1G). This is expected as TSPYL1sh#2 targets the 3’UTR of the endogenous transcripts which is absent in the ectopic cDNA, while TSPYL1sh#4 targets the TSPYL1 coding region in both endogenous and ectopic transcripts. Taken together, our results confirmed that TSPYL1 depletion drives EMT in BE(2)‐C and A549 cells. We conclude that TSPYL1 suppresses EMT.

**Figure 2:**
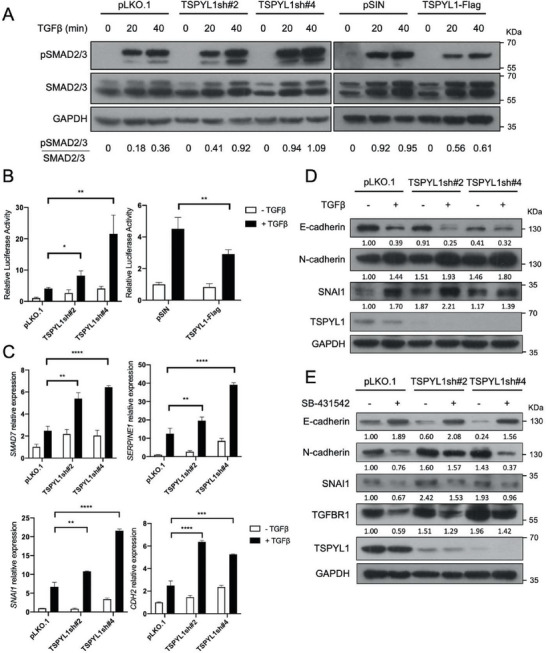
TSPYL1 knockdown promotes TGFβ driven EMT in A549 cells. A) Phospho‐SMAD2/3 protein levels were measured upon TGFβ treatment by immunoblotting. Cells were transduced with viruses as indicated on top for 48 h, and experiments were performed 3 days post‐transduction in all subsequent experiments. A dose of 5 ng mL^−1^ TGFβ1 was added to the culture medium and protein lysates were collected at 0, 20, and 40 min for immunoblotting. B) TGFβ driven transcriptional activities as measured by a luciferase reporter containing 4 SMAD binding elements. TSPYL1 knockdown or overexpression A549 cells were transfected with reporter plasmids for 24 h, serum starved for 24 h and 2 ng mL^−1^ TGFβ1 was added to culture medium for 24 h before luciferase reporter assay. Data are shown as the mean ± S.D. *n* ≥ 3, ^*^ *p* < 0.05, ^**^ *p* < 0.01 by unpaired Student's *t*‐test. C) The transcript levels of TGFβ target genes and mesenchymal marker gene *CDH2* were measured by qPCR. Control and knockdown cells were serum starved for 24 h and treated with or without 5 ng mL^−1^ TGFβ1 for 24 h. The mRNA levels of indicated genes were analyzed by qPCR. *n* = 3, ^**^ *p* < 0.01, ^***^ *p* < 0.001, ^****^ *p* < 0.0001 by unpaired Student's *t*‐test. D) The expression of EMT markers was analyzed by immunoblotting upon TGFβ treatment. Control and knockdown cells as indicated on top were serum starved for 24 h and then treated with or without 5 ng mL^−1^ TGFβ1 for another 24 h for immunoblotting. E) The expression of EMT markers and TGFBR1 were analyzed by immunoblotting. Control and knockdown cells as indicated were treated with 10 µm TGFBR1 inhibitor SB‐431542 for 72 h and samples were immunoblotted for proteins as indicated.

TGFβ is one of the major inducers of EMT.^[^ ^15^ ^]^ We reason that TSPYL1 controls the sensitivity to TGFβ and TSPYL1 KD results in the EMT phenotype due to upregulation of TGFβ signaling. This can be evaluated by the level of pSMAD2/3 and quantification of the luciferase reporter activity driven by the SMAD binding element.^[^ ^16^ ^]^ Upon TGFβ treatment, TSPYL1 KD A549 cells indeed showed greater phosphorylation of SMAD2/3 while OE of TSPYL1 attenuated the level of pSMAD2 (**Figure** **2**A). Consistently, TSPYL1 KD cells had higher reporter activities upon stimulation by TGFβ while TSPYL1 OE cells had lower activities (Figure 2B). TGFβ activates the transcription of direct target genes *SMAD7*,^[^ ^17^ ^]^ *SERPINE1*,^[^ ^18^ ^]^ and *SNAI1* via SMAD proteins.^[^ ^19^ ^]^ In turn, EMT transcription factors such as SNAI1 change the expression of epithelial and mesenchymal genes.^[^ ^15a^ ^]^ Under TGFβ treatment, TSPYL1 KD induced a much higher fold change in the transcript level of the above three TGFβ direct target genes and mesenchymal gene *CDH2* (Figure 2C) as well as the protein level of EMT markers (Figure 2D). Furthermore, the specific TGFBR1 kinase inhibitor SB‐431542 prevented the changes of EMT markers in TSPYL1 KD cells (Figure 2E). Therefore, our results verify that TSPYL1 represses TGFβ signaling and TGFβ driven EMT.

### 2.2 TSPYL1 Binds Gene Promoters in TGFβ Signaling Pathway Relevant to EMT

**Figure 3:**
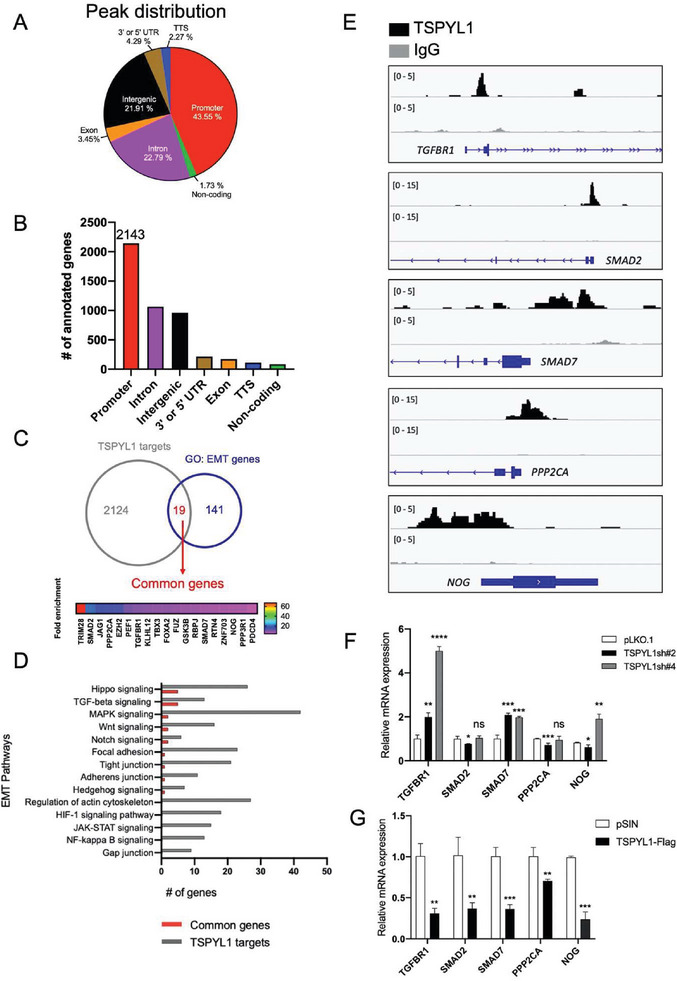
TSPYL1 binds TGFβ pathway genes in EMT in A549 cells. A) Genomic distribution of TSPYL1 peaks identified by CUT&RUN‐next generation sequencing. Promoter: ‐2000 bp to +100 bp of transcription start site; TTS: Transcription Termination Site; UTR: Untranslated Region. B) The number of annotated genes from TSPYL1 CUT&RUN peaks according to HOMER Version 4.9 (http://homer.ucsd.edu/homer/). C) Venn diagram comparing TSPYL1 target genes with EMT genes. The TSPYL1 targets were defined as genes with CUT&RUN peaks in the promoter region. The EMT gene list was from GeneOntology database (GO: 0001837, http://amigo.geneontology.org/). The fold enrichment of peaks in 19 common genes between TSPYL1 targets and EMT genes were indicated in the bar chart. D) TSPYL1 targets were subjected to KEGG pathway analysis and the EMT related pathways were selected. The number of genes in published pathways related to EMT was shown. The number of common genes with the GO EMT gene list is indicated in red. E) Genomic binding of TSPYL1 in the promoter region of TGFβ pathway EMT genes as shown by integrated genomic view. Peak tracks of IgG (light grey) served as the negative control. Scale indicated in the bracket on the left. F, G) Transcript levels of TGFβ pathway genes in A549 cells transduced with control and TSPYL1 knockdown (F) or overexpression viruses (G). Data are mean ± SD, *n* = 3, ^*^ *p* < 0.05, ^**^ *p* < 0.01, ^***^ *p* < 0.001, ^****^ *p* < 0.0001, ns: not significant, by unpaired Student's *t*‐test.

As TSPYL1 contains a NAP domain, we have tested the hypothesis that TSPYL1 is part of the transcription regulatory complex in TGFβ pathway gene promoters. We first confirmed that TSPYL1 was associated with the chromatin (Figure S4A, Supporting Information) with the chromatin purification method.^[^ ^20^ ^]^ We also made use of our KO cells to confirm the specificity of TSPYL1 antibodies by immunocytochemistry (Figure S4B, Supporting Information) for subsequent Cleavage Under Targets and Release Using Nuclease (CUT&RUN) experiment. Next, we examined TSPYL1 genomic localization by CUT&RUN followed by Next‐generation sequencing in A549 cells. In agreement with the model that TSPYL1 regulates gene expression directly, a majority (43.55%) of peaks mapped to the promoter region (here defined as ‐2000 bp to + 100 bp) (**Figure** **3**A). The peaks in the promoter region were assigned to 2143 annotated genes (Figure 3B). Of these, 19 genes are amongst the 160 EMT genes listed under the GeneOntology database (GO: 0001837, http://amigo.geneontology.org/) (Figure 3C). From KEGG pathway analysis, 13 out of the total 2143 genes are components of TGFβ signaling, while 5 of the 19 EMT genes are involved in both TGFβ signaling and published EMT pathways (Figure 3D).^[^ ^15a^ ^]^ The TSPYL1 binding peaks in these 5 genes, namely *TGFBR1*, *SMAD2*, *SMAD7*, *PPP2CA* and *NOG*, were shown (Figure 3E). We further checked if the transcript levels of these 5 genes were affected upon TSPYL1 KD. Only *TGFBR1* and *SMAD7* showed a consistent and significant increase in transcript levels upon KD (Figure 3F), while TSPYL1 OE significantly reduced the expression of all 5 genes (Figure 3G). These data pointed toward the importance of TSPYL1 in directly suppressing *TGFBR1* and *SMAD7*.

### 2.3 TSPYL1 Interacts with FOXA1 to Repress TGFBR1

**Figure 4:**
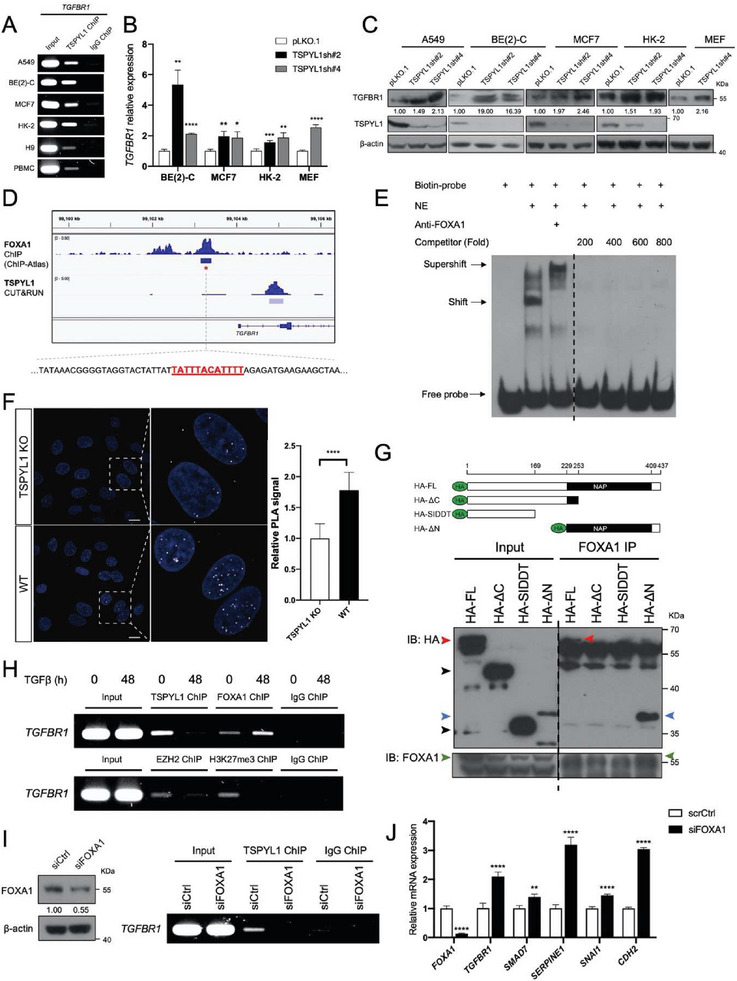
TSPYL1 interacts with FOXA1 to bind the promoter of *TGFBR1*. A) Binding of TSPYL1 to *TGFBR1* promoter in indicated cell lines or cells as verified by ChIP‐PCR. IgG was used as the negative control. PBMC: peripheral blood mononuclear cells. B, C) Increased transcripts (B) and protein (C) of TGFBR1 in TSPYL1 knockdown cells. At 3 days post‐transduction, cells were subjected to qPCR and immunoblotting. MEF: mouse embryonic fibroblasts. Data are shown as the mean ± SD, *n* ≥ 3, ^*^ *p* < 0.05, ^**^ *p* < 0.01, ^***^ *p* < 0.001, ^****^ *p* < 0.0001 by unpaired Student's *t*‐test. D) Visualization of FOXA1 ChIP‐seq profile within the genomic region surrounding the promoter of *TGFBR1* from the website (http://chip‐atlas.org). The binding motif of FOXA1 (in red) was predicted with MoLoTool (https://molotool.autosome.org/). TSPYL1 CUT&RUN peaks were placed alongside for reference. E) EMSA showing shift in the predicted FOXA1 binding motif‐containing oligos (lane 2) and supershift in the presence of FOXA1 antibodies (lane 3). NE: nuclear protein extract from A549; Competitor: unlabeled oligos. F) Confocal images showing the endogenous TSPYL1‐FOXA1 interaction in A549 wildtype (WT) cells as analyzed by proximity ligation assay (PLA). White punctate staining indicated the interaction between TSPYL1 and FOXA1 within 40 nm and DAPI was used to reveal the nucleus. The quantification of positive signal relative to TSPYL1 knockout (KO) A549 cells (negative control) was shown on the right. Data are shown as the mean ± SD, *n* ≥ 10 images, ^****^ *p* < 0.0001 by unpaired Student's *t*‐test. Scale bar: 10 µm. G) Co‐immunoprecipitation to determine the region of TSPYL1 for interacting with FOXA1. The upper panel is the schematic of full length TSPYL1 and its truncated versions. FL: Full length; ΔC: C‐terminal truncated; SIDDT: Truncated protein from a frame shift allele in Sudden Infant Death with Dysgenesis of Testes syndrome. HA‐tagged TSPYL1 FL and its truncation plasmids were transiently transfected into HEK293FT cells. At 72 h post‐transfection, cells were subjected to immunoprecipitation (IP) with FOXA1 antibodies and immunoblot (IB) with indicated antibodies. H) ChIP‐PCR showed the dynamic binding of TSPYL1, FOXA1, EZH2; and H3K27me3 mark at *TGFBR1* promoter under TGFβ treatment. A549 cells were treated with or without 5 ng mL^−1^ TGFβ1 for 48 h and subjected to ChIP with TSPYL1, FOXA1, EZH2, H3K27me3 and IgG antibodies. *TGFBR1* promoter fragments were amplified by PCR. I) ChIP‐PCR showed the loss of TSPYL1 binding upon FOXA1 knockdown. A549 cells transfected with control siRNA (siCtrl) or siRNA targeting FOXA1 (siFOXA1) for 48 h and subjected to ChIP with TSPYL1 and IgG antibodies. *TGFBR1* promoter fragments were amplified by PCR. FOXA1 knockdown was verified by immunoblotting on the left. J) FOXA1 knockdown increased the transcript level of *TGFBR1*, TGFβ target genes and mesenchymal marker gene *CDH2*. A549 cells were transfected with siCtrl or siFOXA1 and subjected to qPCR at 48 h post‐transfection. Data are shown as the mean ± SD, *n* = 4, ^**^ *p* < 0.01, ^****^ *p* < 0.0001, by unpaired Student's *t*‐test.

We focus on TGFBR1 since its cell surface abundance is crucial in controlling the cellular sensitivity to TGFβ.^[^ ^21^ ^]^ We first identified suitable antibodies for ChIP in human cells by ChIP‐immunoblotting and further validated the antibody specificity with KO cells (Figure S5A,B, Supporting Information). We observed the binding of TSPYL1 to the promoter of *TGFBR1* in various cell lines, including A549, BE(2)‐C, MCF7, non‐transformed renal tubular cell line HK‐2, human embryonic stem cell line H9, and peripheral blood mononuclear cells from a healthy donor (**Figure** **4**A). Furthermore, TSPYL1 KD resulted in increased expression of *TGFBR1* in A549, BE(2)‐C, MCF7, and HK‐2 (Figure 3F, 4B,C). Although we were unable to find suitable antibodies for detecting mouse TSPYL1, we successfully knocked down *Tspyl1* in primary mouse embryonic fibroblasts (MEF) with TSPYL1sh#4 (Figure S2, supporting information), which led to increased *Tgfbr1* expression (Figure 4B,C) and impaired cell proliferation (Figure S2, Supporting Information). The findings indicate that TSPYL1 directly represses TGFBR1 expression in both normal and cancerous contexts.

The recruitment of TSPYL1 to target promoters is anticipated to require transcription factor partners that recognize specific DNA sequences. We, therefore, utilized the ChIP‐atlas database (http://chip‐atlas.org/) to search for transcription factors that experimentally bind ± 5 kb of *TGFBR1* transcription start site in A549 cells. Our analysis identified 11 transcription factors including FOXA1, FOSL2, JUN, NR3C1, USF1, MAX, CTCF, E2F6, CEBPB, JUNB and SMAD3. Among them, the loss of FOXA1 has been shown to drive EMT in various cancers.^[^ ^22^ ^]^ To further investigate the role of FOXA1 in TGFBR1 regulation, we searched within the FOXA1 ChIP peak using the Sequence Motif Location Tool (https://molotool.autosome.org/). This analysis identified the putative FOXA1‐binding site located at ‐658 to ‐647 bp from the *TGFBR1* transcription start site (Figure 4D). The supershift assay confirmed that this predicted site is a bona fide FOXA1 binding site (Figure 4E). We further searched the ChIP‐atlas database for other FOXA1 target genes in A549 cells that overlap with TSPYL1 target genes identified in our current CUT&RUN and identified 586 genes that are common targets of both FOXA1 and TSPYL1 (Figure S6A,B; Table S1, Supporting Information).

Next, we tested whether TSPYL1 co‐operates with FOXA1 in vivo. Co‐localization between these endogenous proteins was detected by immunocytochemistry (Figure S7A, Supporting Information) and further demonstrated by Proximity Ligation Assay (PLA), which detects protein molecules located within 40 nm (Figure 4F). To map the domain in TSPYL1 for interaction with FOXA1, we constructed the full‐length HA‐tagged TSPYL1 and its truncated versions. Only the full‐length TSPYL1 and the ΔN version containing the full NAP domain and C‐terminus interacted with FOXA1, while the truncation causing SIDDT did not (Figure 4G; Figure S7B, Supporting Information).

To gain insights into the dynamics of TSPYL1/FOXA1 complex binding to the *TGFBR1* promoter during EMT, we treated A549 cells with TGFβ. Intriguingly, FOXA1 but not TSPYL1 remained bound to the *TGFBR1* promoter after 48 h of TGFβ treatment (Figure 4H). FOXA1 protein stability is enhanced through methylation by EZH2,^[^ ^23^ ^]^ which also serves as the methyltransferase for the repressive histone 3 mark H3K27me3. Previous studies have reported that H3K27me3 is associated with the suppression of *TGFBR1* transcription.^[^ ^24^ ^]^ In agreement with this, we detected the presence of EZH2 binding and the H3K27me3 mark before the addition of TGFβ but not afterward (Figure 4H). Furthermore, TSPYL1 binding was abolished upon FOXA1 KD (Figure 4I), which resulted in the loss of transcriptional repression of *TGFBR1* and several TGFβ target genes including *SMAD7*, *SERPINE1*, and *SNAI1*, as well as SNAI1 target gene *CDH2* (Figure 4J). Taken together, the repression of *TGFBR1* by TSPYL1 is dependent on FOXA1. The proximity of TSPYL1, FOXA1, EZH2, and H3K27me3 to the *TGFBR1* promoter (Figure 4D,H) further confirms that TSPYL1 represses *TGFBR1* through partnering with FOXA1 and EZH2.

### 2.4 TSPYL1 Knockdown Stabilizes TSPYL2 through TGFβ Signaling

**Figure 5:**
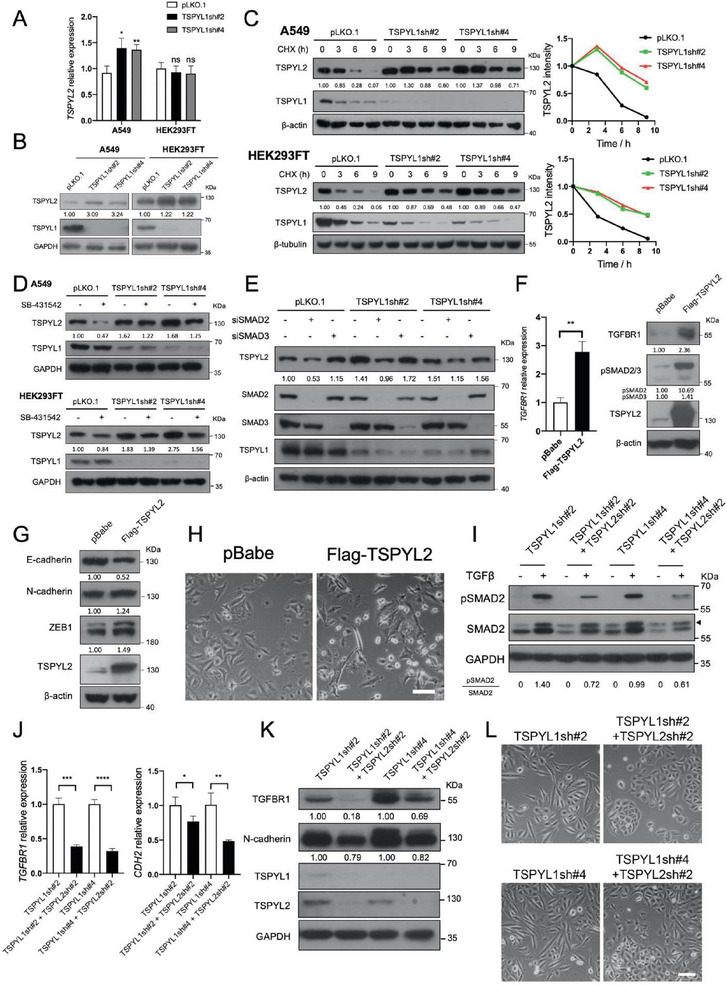
Stabilization of TSPYL2 upon TSPYL1 knockdown mediates TGFβ signaling and EMT. A) *TSPYL2* transcripts increased upon *TSPYL1* knockdown in A549 but not in HEK293FT cells. Cells were infected by lentivirus and subjected to qPCR three days after infection. Data are shown as the mean ± SD, *n* = 3, ^*^ *p* < 0.05, ^**^ *p* < 0.01, ns: not significant by unpaired Student's *t*‐test. B) TSPYL2 protein increased upon TSPYL1 knockdown in A549 and HEK293FT cells. Cells were infected by lentivirus and subjected to immunoblotting 3 days after transduction. C) TSPYL2 protein was stabilized upon TSPYL1 knockdown. Three days after infection, control and TSPYL1 knockdown cells were treated with cycloheximide (CHX; 50 µg mL^−1^ for A549, 10 µg mL^−1^ for HEK293FT cells) for the indicated time. Immunoblotting signals of TSPYL2 to loading control were quantified with image J and normalized to the ratio at 0 h treatment. Normalized signals were indicated and plotted in graph on the right. D) The upregulation of TSPYL2 upon TSPYL1 knockdown was mitigated by the inhibition of TGFβ signaling in A549 and HEK293FT cells. After infection, cells were treated with DMSO or 10 µm SB‐431542 for 72 h (A549) or 24 h (HEK293FT) and subjected to immunoblotting. E) The upregulation of TSPYL2 upon TSPYL1 knockdown was abolished by knockdown of SMAD2 but not SMAD3. A549 cells were infected with lentiviruses for 24 h to knockdown TSPYL1, followed by transfection with siRNA targeting SMAD2 or SMAD3 for 24 h. After transfection, cells were cultured for another 36 h and subjected to immunoblotting. F) Three days after transduction with pBabe or Flag‐TSPYL2 in A549 cells, RNA and protein samples were collected for measuring the transcript level of *TGFBR1* (left) or immunoblotting for the indicated proteins (right). Data are mean ± SD, *n* = 3. ^**^ *p* < 0.01, unpaired Student's *t*‐test. G) Seven days after transduction with pBabe or Flag‐TSPYL2 in A549 cells, the protein level of epithelial marker E‐cadherin and mesenchymal markers as indicated were determined by immunoblotting. H) Representative images of control (pBabe) and Flag‐TSPYL2 A549 cells seven days after transduction. Scale bar: 50 µm. I–L) Rescue of TSPYL1 knockdown‐induced EMT phenotype by co‐knockdown of TSPYL2. A549 cells were infected with viruses as indicated. I) 3 days after infection, cells were treated with 5 ng mL^−1^ TGFβ1 for 40 min and subjected to immunoblotting. The protein was quantified with Image J to indicate reduced pSMAD2 upon double knockdown. J,K) RNA and protein samples were collected 5 days after infection for qPCR and immunoblotting. Data are shown as the mean ± SD, *n* = 3, ^*^ *p* < 0.05, ^**^ *p* < 0.01, ^***^ *p* < 0.001, ^****^ *p* < 0.0001 by unpaired Student's *t*‐test. L) Images under bright field were taken 6 days after infection. Scale bar: 50 µm.

TSPYL2 is upregulated by the addition of TGFβ in certain cell lines and in mouse primary aortic smooth muscle cells.^[^ ^8^, ^25^ ^]^ Therefore, we examined whether TSPYL2 is elevated upon TSPYL1 KD via increased TGFβ signaling. Even though there was only a mild increase or no change in transcript level, TSPYL2 protein levels were significantly increased upon TSPYL1 KD in both A549 and HEK293FT, indicating enhancement of protein stabilization (**Figure** **5**A,B). TSPYL2 is subjected to proteasome degradation.^[^ ^26^ ^]^ To further verify the enhanced TSPYL2 stability, we treated A549 and HEK293FT cells with cycloheximide (CHX) to inhibit protein synthesis and examined the half‐life of TSPYL2 in the control and TSPYL1 KD environment. Indeed, the rate of TSPYL2 protein reduction by 50% in TSPYL1‐depleted cells was slower than that of vector control (Figure 5C). Moreover, inhibition of TGFBR1 kinase activity prevented the up‐regulation of TSPYL2 in TSPYL1 KD A549 and HEK293FT cells, confirming that TSPYL2 upregulation is dependent on TGFβ signaling in both cell lines (Figure 5D). TSPYL2 stabilization was impaired in TSPYL1 KD cells when SMAD2, but not SMAD3 was KD (Figure 5E), indicating the dependence on the canonical SMAD pathway. In addition, OE of TSPYL2 increased transcript and protein levels of *TGFBR1* and increased phosphorylation of SMAD2/3 (Figure 5F), in relation to increased expression of mesenchymal markers and reduced epithelial marker E‐cadherin upon further culture until morphological changes similar to EMT were observable (Figure 5G,H). To prove that TSPYL2 mediates the EMT phenotype in TSPYL1 KD, co‐KD of TSPYL1 and TSPYL2 was employed for the rescue experiments. Criteria for success include the attenuation of TGFβ induced SMAD2 phosphorylation (Figure 5I) and expression of *TGFBR1* and *CDH2* (Figure 5J,K). Importantly, cells regained the cobblestone morphology (Figure 5L). The data demonstrate that TSPYL2 stabilization is caused by TGFβ‐SMAD2 signaling and leads to EMT in A549 cells.

### 2.5 TSPYL2 is an Effector of TGFβ Signaling

**Figure 6:**
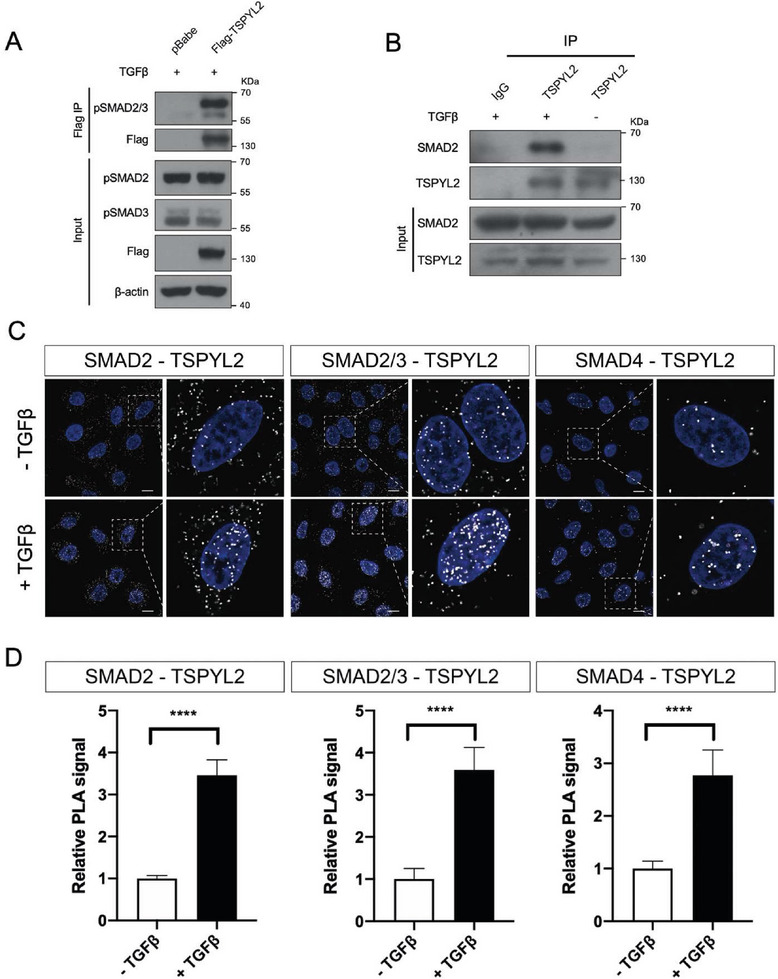
TSPYL2 mediates TGFβ transcriptional responses in A549 cells. A) Phosphorylated SMAD2/3 complex co‐immunoprecipitated with Flag‐TSPYL2 under TGFβ treatment. The control and Flag‐TSPYL2 transduced A549 cells were treated with 5 ng mL^−1^ TGFβ1 for 1 h and subjected to immunoprecipitation (IP) with Flag beads and immunoblotted for the indicated protein. B) Endogenous TSPYL2 immunoprecipitated with SMAD2 under TGFβ treatment. A549 cells were treated with or without 5 ng mL^−1^ TGFβ1 for 1 h and subjected to IP with antibodies to TSPYL2 or IgG as indicated on the top and immunoblotted for proteins as indicated on the left. C) Interaction between TSPYL2 and SMAD2/3/4 complex upon the addition of TGFβ by proximity ligation assay (PLA). A549 cells were treated with or without 5 ng mL^−1^ TGFβ1 for 1 h. White punctate staining showed the interaction between TSPYL2 and components of SMAD complex as indicated on the top and DAPI was used to reveal the nucleus. Scale bar: 10 µm. D) Quantitation of punctate staining after TGFβ treatment relative to no TGFβ treatment group. Data are shown as the mean ± SD, *n* = 5 images, ^****^ *p* < 0.0001 by unpaired Student's *t*‐test.

**Figure 7:**
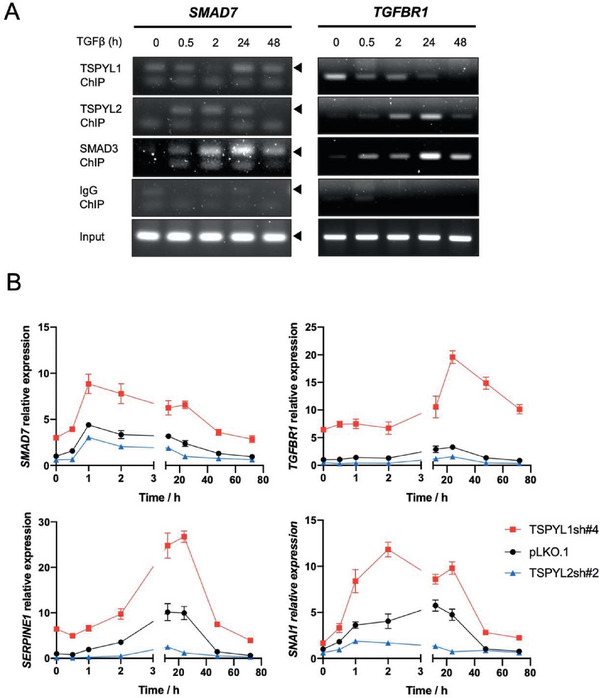
TSPYL1 and TSPYL2 oppositely regulate TGFβ target genes in A549 cells. A) ChIP‐PCR showing the time course of binding of TSPYL1, TSPYL2 and SMAD3 to *SMAD7* and *TGFBR1* promoters upon stimulation by TGFβ. A549 cells were treated with 5 ng mL^−1^ TGFβ1 for the indicated time and subjected to ChIP with indicated antibodies. IgG was used as the negative control. B) The effects of TSPYL1 or TSPYL2 knockdown on transcriptional responses to TGFβ were investigated by qPCR. A549 cells were treated with 5 ng mL^−1^ TGFβ1 for the indicated time on x‐axis before sample collection at 7 days post‐transduction. Data are shown as the mean ± SD, *n* = 4.

To refine the role of TSPYL2 in TGFβ signaling, we asked if TSPYL2 is part of the SMAD complex following the addition of TGFβ to regulate transcription. We overexpressed Flag‐TSPYL2 (gift from Dr. Pier Paolo Pandolfi) for immunoprecipitation with anti‐Flag conjugated beads to allow clear detection of pSMAD2/3. Preferential binding of TSPYL2 to pSMAD2 was detected (**Figure** **6**A). For endogenous TSPYL2, co‐immunoprecipitation with SMAD2 was detected only upon the addition of TGFβ (Figure 6B). To allow more sensitive detection of transient interaction, PLA was employed to detect the interaction between TSPYL2 and components of SMAD2/3/4 complex according to the availability of suitable antibodies. Signals were detected under the basal condition in both the cytoplasm and nucleus. As expected, there was a significant increase in nuclear signals upon the addition of TGFβ (Figure 6C,D). The abundant signals in the cytoplasm also clearly indicated that TSPYL2 interacted with the SMAD complex before nuclear import. Next, we tested whether TSPYL2 works with SMAD2/3/4 to regulate the transcription of *SMAD7*, a well‐known direct target gene of TGFβ signaling.^[^ ^17^ ^]^ Since TSPYL1 was not detected at the *TGFBR1* promoter at 48 h of TGFβ treatment (Figure 4H) and OE of TSPYL2 caused increased TGFBR1 (Figure 5F), we also checked whether TSPYL2 binds to the *TGFBR1* promoter. In agreement with the notion that TSPYL2 is part of the SMAD2/3/4 signal transducer, TSPYL2 was already detected at the *SMAD7* promoter 0.5 h after the addition of TGFβ. Importantly, the binding of TSPYL2 to the *TGFBR1* promoter increased from 0.5 to 24 h after the addition of TGFβ and showed the same binding kinetics with SMAD3. TSPYL1 binding was weak when the binding of TSPYL2 prevailed (**Figure** **7**A). The binding patterns show that TSPYL1 and TSPYL2 regulate these two TGFβ pathway component genes in the opposite manner by alternative binding to the promoter.

Finally, we verified the importance of TSPYL1 and TSPYL2 in regulating TGFβ target genes by measuring their transcript levels in TSPYL1 or TSPYL2 KD cells. As expected, there was an early increase in *SMAD7* and a delayed increase in *TGFBR1* transcript levels when TGFβ was added in control or KD cells. Furthermore, the ground level of all four tested genes including also *SERPINE1* and *SNAI1* was elevated in TSPYL1 KD cells, which agreed to increased TGFβ signaling in TSPYL1 KD cells. By contrast, there was an impaired transcriptional response to TGFβ in TSPYL2 KD cells (Figure 7B). Together, the data support the notion that TSPYL1 and TSPYL2 play a counter balancing role in the transcription of TGFβ target genes. TSPYL1 interacts with FOXA1 to suppress TGFβ signaling at basal state while TSPYL2 interacts with the SMAD complex to promote it upon stimulation.

### 2.6 TSPYL1 Regulates TGFβ Signaling In Vivo

**Figure 8:**
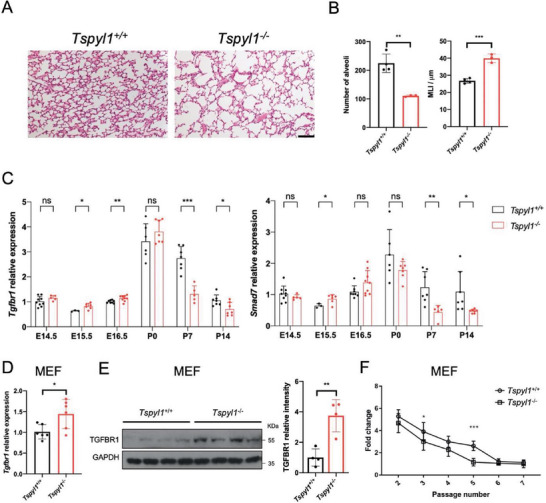
*Tspyl1* knockout mice have mis‐regulated TGFβ signaling and associated phenotype. A) Representative lung sections from wildtype (*Tspyl1^+/+^)* and knockout (*Tspyl1^−/−^)* mice at postnatal day 14. Lungs were inflated before fixation and sections were stained with hematoxylin and eosin. Scale bar: 100 µm. B) Morphometric analysis of sections collected as above. Images were digitally captured at 20X magnification for determining the number of alveoli and mean liner intercept (MLI). Data are shown as the mean ± SD, *n* = 4 wildtype and 3 knockout lungs. ^**^ *p* < 0.01, ^***^ *p* < 0.001 by unpaired Student's *t*‐test. C) qPCR of lung samples collected at the indicated time points. Day of birth was designated as P0. Data are shown as the mean ± SD, *n* = 3–9, ^*^ *p* < 0.05, ^**^ *p* < 0.01, ^***^ *p* < 0.001, ns: not significant by unpaired Student's *t*‐test. D,E) Expression of TGFBR1 in wildtype and knockout primary mouse embryonic fibroblasts (MEF). MEF were prepared from wildtype and *Tspyl1* knockout littermates and collected at passage 3 for (D) qPCR, *n* = 6 per genotype and (E) immunoblotting, *n* = 4 per genotype. Data are shown as the mean ± SD, ^*^ *p* < 0.05, ^**^ *p* < 0.01 by unpaired Student's *t*‐test. F) Early replication arrest in *Tspyl1* knockout MEF. Following the 3T3 protocol, MEF collected from wildtype and knockout littermate embryos were seeded at a density of 3 × 10^5^ on 5 cm plates (passage 1). The number of cells was counted every 3 days and re‐seeded at the same density. The fold change was calculated by dividing the cell number with 3 × 10^5^ at each passage. Fold change of one indicates replication arrest. Data are shown as the mean ± SD, *n* = 6 per genotype, ^*^ *p* < 0.05, ^***^ *p* < 0.001, two‐way ANOVA.

In mice, TGFβ is important during branching morphogenesis and late lung development.^[^ ^27^ ^]^ In neonates, the respiratory surface is greatly increased by subdivision of alveoli by septa which reduces the size but increases the number of alveoli. Either under or excess TGFβ signaling resulted in impaired alveolar septation.^[^ ^27^, ^28^ ^]^ Since most *Tspyl1* KO mice die ≈15 days after birth, we investigated whether there was any defect in neonatal lung development. From histological studies, there was indeed a septation defect in *Tspyl1* KO mice as indicated by a reduced number of alveoli which were of larger sizes (**Figure** **8**A,B). We further examined whether there was perturbed expression of *Tgfbr1* and *Smad7* in KO lungs. We detected upregulated transcript levels at embryonic day 15.5 and 16.5 (pseudoglandular stage), and significant downregulation at postnatal day 7 and day 14 (alveolar stage; Figure 8C). In addition, we collected wildtype and KO MEF in litters derived from *Tspyl1* heterozygous parents. *Tspyl1* KO MEF consistently showed elevated expression of *Tgfbr1* transcript and protein (Figure 8D,E), which was associated with reduced proliferation and earlier replication arrest at passage 5 (Figure 8F). The data support a critical role of TSPYL1 in regulating the expression of *Tgfbr1* in vivo.

## 3 Discussion

This study shows that TSPYL1 is a critical negative regulator of TGFβ signaling and EMT in cell models. In general, TSPYL1 binds the promoter of *TGFBR1* to refine the cellular response to TGFβ. It suppresses the function of the closely related TSPYL2 which can act as a signal transducer of TGFβ. Mechanistically, TSPYL1 partners with FOXA1 and EZH2 to repress *TGFBR1* to maintain the cellular level of TGFβ receptors. In both lung carcinoma A549 and neuroblastoma BE(2)‐C cells, TSPYL1 depletion causes EMT. The underlying mechanism involves the upregulation of TGFβ signaling via elevated transcription of *TGFBR1*. Upon stimulation by TGFβ, TSPYL2 forms a complex with SMAD2/3/4 to regulate TGFβ target genes. The changes in gene expression upon activation by TGFβ follow the switch of *SMAD7* and *TGFBR1* promoter occupancy by TSPYL1 and TSPYL2. Our findings underscore the counterbalancing role of TSPYL1 and TSPYL2 as transcription regulators of TGFβ signaling. The upregulation of TSPYL2 in TSPYL1 KD cells and genetic rescue by co‐knockdown of TSPYL1 and TSPYL2 provide insights that perturbed TGFβ signaling and TSPYL2 stabilization may be the underlying disease mechanism in SIDDT.

TGFβ signaling has pleiotropic effects in both normal development and disease processes.^[^ ^2^ ^]^ Depending on the cellular context, it controls cell proliferation, survival, and migration. In our study, we uncover that TSPYL1 binds the promoter of *TGFBR1* in a variety of cancer cell lines as well as normal cells. We also observed that *TSPYL1* depletion adversely affected the proliferation of all cell lines being tested and in MEF. Furthermore, besides *TGFBR1*, *SMAD7* is also a direct TSPYL1 target. Both genes are pivotal in determining the cellular sensitivity to TGFβ signaling. Upon addition of TGFβ, the intracellular level of TGFBR1 determines the availability of cell surface receptors; while SMAD7 provides the negative feedback on TGFβ signaling.^[^ ^29^ ^]^ Our TSPYL1 KD and OE studies point toward the role of TSPYL1 in repressing the transcription of these two TGFβ signaling component genes in cell models. In other words, TSPYL1 is essential to maintain the basal levels of *TGFBR1* and *SMAD7*. TSPYL1 is ubiquitously expressed. TSPYL1 might repress various TGFβ target genes through forming the FOXA1/EZH2 complex in cell types with FOXA1 expression. Candidates can be considered from the list of overlapping TSPYL1/FOXA1 target genes for future studies (Table S1, Supporting Information).

Does TSPYL1 regulate TGFβ signaling in vivo? We have investigated whether there is perturbed TGFβ signaling in *Tspyl1* KO lungs as proper TGFβ and BMP signaling is important in alveolar development.^[^ ^30^ ^]^ We found initially up and then down‐regulation of *Tgfbr1* and *Smad7* transcripts in the mutant lung. This could be due to the presence of heterogeneous cell types and a complex in vivo environment that encompasses various factors such as ligand availability, signaling cross‐talks, and compensatory mechanisms to modulate the cellular response to the absence of *Tspyl1*. Nevertheless, evidence that our in vitro model also holds in vivo was gained from the upregulated *Tgfbr1* and impaired proliferation in both *Tspyl1* KO and in vitro KD MEF.

Our data indicates that TSPYL2 forms part of the SMAD2/3/4 complex upon activation by TGFβ and *TGFBR1* is also a TSPYL2 direct target gene. As part of the SMAD signaling complex, TSPYL2 may be transiently protected from proteasomal degradation upon stimulation by TGFβ. Since induction of growth arrest can reduce MDM2‐mediated ubiquitination of TSPYL2,^[^ ^26a^ ^]^ TSPYL2 may be further stabilized through secondary events of cell proliferation arrest caused by TGFβ. Intriguingly, *TSPYL1* KD stabilizes TSPYL2 in all cell lines we have tested including A549, HEK293FT, BE(2)‐C, MCF7, cervical cancer HeLa and melanoma A375 (Figure 5B,C; Figure S8, Supporting Information). We have only proved that this is dependent on TGFBR1 in A549 and HEK293FT. Our future work is to explore whether TGFβ related or other mechanisms exist to stabilize TSPYL2 upon TSPYL1 depletion in different cellular contexts. Knowledge of the various mechanisms to stabilize TSPYL2 will provide understanding on how TSPYL2 helps the cell to cope with stresses such as DNA damage.^[^ ^31^ ^]^ Independent studies have established that ectopic expression or induction of TSPYL2 by various stresses induces growth arrest or apoptosis in cancer stem cells, cancer cell lines, human embryonic lung fibroblasts, and human mesenchymal stem cells.^[^ ^7^, ^26^, ^32^ ^]^ It is possible that stabilized TSPYL2 and the negative effects on cell proliferation and differentiation may be the caveats in SIDDT. An effective peptide inhibitor for blocking TSPYL2 signaling axis in renal fibrosis has been developed.^[^ ^9^ ^]^ The efficacy of this drug in alleviating the disease process in *Tspyl1* KO mice, due to altered TGFβ or other cellular pathways, should be tested in the future. Finally, our CUT&RUN data also revealed plenty of potential TSPYL1 target genes in the BMP, Hippo, Wnt, Notch, and MAP kinase pathways which are very important in development, homeostasis, and disease. These are all potential mechanisms to explore in order to decipher the biological importance of TSPYL1.

## 4 Conclusion

This study establishes the essential roles of TSPYL1 and TSPYL2 in controlling the expression of key components in the TGFβ signaling pathway. Specifically, expression of both the upstream regulator *TGFBR1* and the negative feedback messenger *SMAD7* are maintained by TSPYL1 in cell models. TSPYL1 deficiency augments TGFβ signaling, which is mediated through the stabilization of TSPYL2 as part of the signal transducer to regulate target gene expression. The findings provide mechanistic insights into the etiology of SIDDT.

## 5 Experimental Section

### Animals

All animal protocols were approved by the Committee on the Use of Live Animals in Teaching & Research at the University of Hong Kong (CULATR 6008–22, 5281‐20). *Tspyl1* KO line Δ463 on pure C57BL/6N background was maintained by breeding between *Tspyl1* ^+/−^ heterozygotes.^[^ ^13^ ^]^

### PBMC isolation

The protocol was approved by the Institutional Review Board (IRB Ref: UW 22–154). PBMC were isolated from buffy coats provided by the Hong Kong Red Cross using Lymphoprep (Thermo Fisher Scientific).

### Materials and Reagents

Plasmids, cloning primers, qPCR primers, ChIP‐PCR primers, and antibodies used in this study were listed in Tables S2–S6 (Supporting Information). TGFβ1 (PHG9214), Pierce Protease Inhibitor Tablets, EDTA‐free (A32965), Halt Protease and Phosphatase Inhibitor Single‐Use Cocktail (100X) (78442), were purchased from Thermo Fisher Scientific, TGFBR1 inhibitor SB‐431542 (HY‐10431) and MG132 (HY‐13259) from MedChemExpress, and protein synthesis inhibitor cycloheximide from Santa Cruz Biotechnology (sc‐3508).

### Cell Culture

BE(2)‐C, A549, MCF7, HeLa, A375, and HK‐2 cells were obtained from the American Type Culture Collection. HEK293FT cells were a gift from Dr. David Wai Chan. H9 cells were a gift from Dr. Cherie Lee and directly used for ChIP. BE(2)‐C, A549, MCF7, HeLa, A375, and HEK293FT were cultured in Dulbecco's Modified Eagle Medium (Invitrogen) supplemented with 10% fetal bovine serum (Hyclone), and HK‐2 cells were cultured in DMEM/F12 (Invitrogen) with 10% fetal bovine serum at 37 °C in 5% CO_2_. All cell cultures were tested negative for mycoplasma.

### MEF Isolation and 3T3 Assay

MEF were isolated from embryos at 13.5 days of gestation as previously described,^[^ ^33^ ^]^ and cultured in Dulbecco's Modified Eagle Medium (Invitrogen) supplemented with 10% fetal bovine serum (Hyclone) and 1% penicillin/streptomycin (Invitrogen) at 37 °C in 5% CO_2_. For knockdown experiments, MEF ‐were pooled from the whole litter derived from wildtype parents. For experiments comparing between wildtype and *Tspyl1* KO, litters were collected from heterozygous parents. MEF derived from each embryo were cultured individually and genotyped with primers as previously described.^[^ ^13^ ^]^ MEF were subcultured using the standard 3T3 protocol. Briefly, 3 × 10^5^ cells were plated on a 5‐cm plate and cultured for 3 days. Cells were trypsinized for counting at day 3 and the same number of cells (3 × 10^5^) were plated again for further passages. The proliferation curve was constructed by dividing the total number of live cells before each passage to the number seeded to give a fold difference.

### Vector Construction

Annealed sgRNAs targeting TSPYL1 (TSPYL1sg#1 targeting sequence: 5’‐AGCGACCAGGACGCACACCA‐3’, and TSPYL1sg#2 targeting sequence: 5’‐ CGGGCCGTGGCGGTACTCCC‐3’) were cloned into the BbsI digested PX459 (gift from Dr. Feng Zhang) for CRISPR/Cas9 mediated gene KO.^[^ ^34^ ^]^ Human TSPYL1 CDS was PCR amplified from the intronless TSPYL1 gene using human genomic DNA and cloned into the EcoRI and XhoI sites of a modified pcDNA3 with HA sequence inserted with indicated primers in Table S2 (Supporting Information). The pcDNA3‐HA‐TSPYL1‐SIDDT plasmid was generated via site‐directed mutagenesis of pcDNA3‐HA‐TSPYL1 with indicated primers in Table S2 (Supporting Information). The truncated pcDNA3‐HA‐TSPYL1 ΔC was generated from pcDNA3‐HA‐TSPYL1 by digestion with PfImI and XhoI, treatment with T4 DNA polymerase, and religation. The truncated HA‐TSPYL1‐ΔN was amplified from pcDNA3‐HA‐TSPYL1 by PCR and ligated into pcDNA3. For stable expression, human TSPYL1 CDS with C‐terminal Flag was subcloned into pSIN (gift from Dr. Qi‐Long Ying) using SpeI and NsiI endonucleases with indicated primers in Table S2 (Supporting Information),^[^ ^35^ ^]^ leading to the formation of pSIN‐TSPYL1‐Flag. The shRNA against human TSPYL2 (targeting sequence: 5’‐CATGGTGATTGTCAAGGAG‐3’) was subcloned into the lentiviral vector pLKO.1‐puro with endonucleases AgeI and EcoRI to generate pLKO.1‐TSPYL2sh#2.

### Generation of Knockout Cells

TSPYL1 KO BE(2)‐C were generated by transfection with PX459‐TSPYL1sg#2. TSPYL1 KO A549 cells were generated by transfection with combined PX459‐TSPYL1sg#1 and PX459‐TSPYL1sg#2. Cells were transfected with lipofectamine 2000 (Thermo Fisher Scientific, 11668019) and selected with 10 µg mL^−1^ puromycin (Thermo Fisher Scientific). Isolated colonies were picked, expanded, and screened for *TSPYL1* KO through immunoblotting. TSPYL1 frameshift mutations were confirmed by Sanger sequencing (Table S7, Supporting Information).

### siRNA Transfection and Lentivirus Transduction

siRNA against FOXA1 (sc‐37930), SMAD2 (sc‐38374) and SMAD3 (sc‐38376) and control siRNA (sc‐37007) were purchased from Santa Curz Technology. siRNA transfections were performed using siRNA Transfection reagent (sc‐29528) with siRNA transfection medium (sc‐36868). Transfections were carried out following the manufacturer's instructions. For preparing the lentivirus medium, 5 × 10^6^ HEK293FT cells were seeded onto a 10‐cm culture plate and incubated at 37 °C with 5% CO_2_ overnight. Lentivirus transfection was conducted with the mixture of 7.5 µg transfer plasmid, 5 µg psPAX2, 2.5 µg pVSVG, and 60 µL 1 µg mL^−1^ PEI in 1 mL OptiMEM (Invitrogen). The transfection complex was incubated for 20 min and added onto HEK293FT cells dropwise. After 24 h, the medium was refreshed. The virus was harvested at 48 and 72 h. For virus collection, the medium from the transfected HEK293FT cells was collected and filtered with a 0.45 µm PES filter. The viral medium was stored at –‐80 °C and thawed at 37 °C before use. The retrovirus medium was prepared with the same protocol except for using pCL‐Ampho as the packaging plasmid rather than psPAX2. For transduction, the viral medium with 8 µg mL^−1^ polybrene (Sigma, TR1003) was added to the target cell lines when they reached ≈50% confluency. Unless otherwise stated, the target cell lines were infected twice with the neat virus medium for 48 h and then cultured with fresh medium for at least 72 h before conducting the experiments.

### Scratch Assay and Transwell Assays

For the scratch assay, cells were grown to confluency. A549 and BE(2)‐C cells were treated with 10 µg mL^−1^ Mitomycin C (Sigma, 50‐07‐7) for 2 and 3 h respectively to stop cell proliferation. Cells were wounded with a 200 µL pipette tip across the cell monolayer, washed, and cultured for another 16 h (A549 cells) or 24 h (BE(2)‐C cells). Phase contrast pictures were taken by an inverted microscope using a magnification of 20X. For the transwell migration assay, 3 × 10^4^ A549 were resuspended in 150 µL complete medium and seeded in the upper chamber of transwells cell culture inserts (Sigma, CLS3464) placed in a 24‐well. A complete medium (600 µL) was placed in the lower chamber. Cells were incubated at 37 °C in 5% CO_2_ for 24 h. Cells that migrated to the lower surface of the transwell were fixed with cold methanol for 10 min, and then stained with 0.1% crystal violet (Sigma, C6158) for 5 min. Non‐invading cells on the upper surface of the membrane were gently removed with cotton tips. The stained cells were counted from images taken under an inverted microscope at a magnification of 20X. In each experiment, the number of stained cells, in at least five independent image fields, were averaged. For the invasion assay, the above‐described procedure of transwell migration assay was conducted, except for the use of transwell inserts previously coated as follows: Matrigel (BD Bioscience, #356234) was diluted 4 times with culture medium. Thirty µL was coated on each transwell insert and allowed to solidify for 2 h in the tissue culture incubator. For BE(2)‐C cells, 1 × 10^5^ cells were resuspended in 100 µL and added to the upper chamber.

### Immunoblotting

Proteins were collected in RIPA buffer (50 mm Tris‐HCl pH 8, 150 mm NaCl, 2 mm EDTA pH 8, 1% NP‐40, 0.5% Sodium Deoxycholate, 0.1% SDS) supplemented with complete protease inhibitors (Thermo Fisher Scientific, A32965). Total proteins were quantitated with Bio‐Rad DC Protein Assay Reagent B. Proteins were resolved by SDS‐PAGE in the Mini‐PROTREAN Tetra Cell system (Bio‐Rad). Proteins were transferred to Nitrocellulose membranes (Bio‐Rad #1620112) using a Mini Trans‐Blot system (Bio‐Rad) at 100 V for 2 h. Membranes were blocked with 5% non‐fat dried milk or 5% BSA (Sigma) in TBS with 0.1% Tween 20 (Sigma) for 1 h. Membranes were incubated in primary antibodies overnight and subsequently washed and incubated in secondary antibodies for 1 h at room temperature. Proteins were visualized using ECL (Thermo Fisher Scientific).

### Immunocytochemistry

Cells were seeded onto coverslips 24 h in advance. Cells were fixed with 4% PFA for 10 min, washed three times with PBS for 5 min, and then blocked with 2% normal goat serum (Thermo Fisher Scientific) in PBST (PBS + 0.1% Tween 20) for 1 h. The primary antibodies in 2% normal goat serum in PBST were added to the cells. After incubation overnight at 4 °C, the cells were washed with PBST three times for 5 min each. The cells then were incubated with the secondary antibodies for 1 h and subsequently washed three times with PBS for 5 min each. To stain the cell nuclei, the cells were incubated with 1 µg mL^−1^ DAPI diluted in 2% normal goat serum in PBS for 5 min. The cells then were washed for 5 min twice with PBST. Finally, the coverslips were mounted onto slides and examined under the confocal microscope (LSM 980 with Airyscan2).

### Luciferase Assay

A549 cells (2 × 10^5^) were seeded onto a 96‐well plate to achieve ≈ 70% confluence on the day of transfection. The Dual‐Luciferase reporter assay system (Promega, E1980) was used according to the manufacturer's protocol. Briefly, a mixture containing Lipofectamine 2000, luciferase reporter plasmid DNA (pGL3‐SBE4‐luc (gift from Dr. Peter ten Diike ^[^ ^16^ ^]^) 100 ng, RLuc 10 ng per well) was added. After 24 h, cells were starved with serum‐free medium for 24 h, followed by another 24 h of treatment with 2 ng mL^−1^ TGFβ1 in complete medium. Subsequently, cells were harvested by Passive lysis buffer, and luciferase activity was measured. Each assay consisted of at least three replicate wells.

### RNA Extraction and Quantitative Real‐Time PCR Analysis (qPCR)

Total RNA was extracted with Rnazol (Molecular Research Center, RN190) following the manufacturer's instructions. Total RNA was used for reverse transcription in 20 µL using oligo(dT) (Thermo Fisher Scientific) and SuperscriptII reverse transcriptase (Thermo Fisher Scientific, 18064022). qPCR was conducted with 0.5 µL of cDNA by using iTAQ Universal SYBR Green Supermix (Bio‐Rad, 1725122) in Roche LightCycler 480 II. *HPRT* was used as the internal control. The ΔΔCt method was used for calculating the relative expression.

### CUT and RUN Assay and Bioinformatics Analysis

CUT&RUN was performed according to the protocol using the high‐calcium/low‐salt digestion conditions with slight modifications.^[^ ^36^ ^]^ All reactions were performed at 4 °C unless stated otherwise. Each centrifugation step was performed at 600 g for 3 min. Cells were washed twice with PBS and resuspended in Buffer NE1 (20 mm HEPES‐KOH, pH 7.9, 10 mm KCl, 0.5 mm Spermidine, 0.1% Triton X‐100, 20% Glycerol, protease inhibitors). After 10 min, cells were collected and incubated in Buffer 1 (20 mm HEPES‐KOH pH 7.9, 150 mm NaCl, 2 mm EDTA, 0.5 mm Spermidine, 0.1% BSA, protease inhibitors) for 5 min. Cells were collected and washed once with Buffer 2 (20 mm HEPES, pH 7.5, 150 mm NaCl, 0.5 mm Spermidine, 0.1% BSA, protease inhibitors) and resuspended in Buffer 2. Samples were incubated with TSPYL1 antibodies (ProteinTech, 13932‐1‐AP) or normal Rabbit IgG (Millipore, PP64B) overnight. Afterward, nuclei were collected and washed twice with Buffer 2 and then incubated with a pA‐MNase fusion protein (150 ng) for 1 h. Nuclei were washed twice with Buffer 2 and once with low‐salt buffer (3.5 mm HEPES, protease inhibitors). Subsequently, samples were resuspended in low‐salt buffer and 10 mm CaCl_2_ was added to each reaction. MNase digestion was stopped after 30 min with the addition of Buffer 2 with 10 mm EDTA, and 20 mm EGTA. The reactions were incubated for another 15 min at 37 °C. The DNA in the supernatant was collected and purified with a QIAQUICK PCR Purification Kit (QIAGEN). Libraries were constructed with KAPA Hyper Prep Kit (KR0961‐V1.14). The libraries were denatured and diluted to the optimal concentration. Illumina NovaSeq 6000 was used for Pair‐End 151 bp sequencing. For the data analysis, the raw sequencing reads were filtered for low‐quality sequences and adapter sequences followed by retaining only reads with read length over 40 bp. The filtered reads were mapped to Human Genome GRCh38 using Bowtie2 (http://bowtie‐bio.sourceforge.net/bowtie2/). The peaks were called using MACS2 (https://github.com/taoliu/MACS). The peaks were annotated with HOMER (http://homer.ucsd.edu/homer/). The KEGG pathway enrichment analysis was done at the Enrichr database (https://maayanlab.cloud/Enrichr/).

### Chromatin Immunoprecipitation (ChIP)

ChIP was performed following the X‐ChIP protocol from Abcam. Briefly, cells were crosslinked with 0.75% formaldehyde (Sigma) in culture medium for 5 min at room temperature, and chromatin from lysed nuclei was sheared into 200–600 bp fragments using Diagenode BioruptorPico Sonicator. Chromatin was immunoprecipitated with antibodies of TSPYL1 (ProteinTech, 13932‐1‐AP), FOXA1(Abcam, ab170933), TSPYL2 (ProteinTech, A304‐012A), SMAD3 (Abcam, ab40854), EZH2 (Cell Signaling Technology, #5246), H3K27me3 (Diagenode, pAb‐069‐050) or normal Rabbit IgG (Millipore, PP64B) overnight at 4 °C. Pre‐washed Protein A agarose beads (Santa Cruz Biotechnology, sc‐2001) were added and then further incubated for 2 h at 4 °C. The samples were washed once with low salt wash buffer (0.1% SDS, 1% Triton X‐100, 2 mm EDTA, 20 mm Tris‐HCl pH 8.0, 150 mm NaCl), once with high salt wash buffer (0.1% SDS, 1% Triton X‐100, 2 mm EDTA, 20 mm Tris‐HCl pH 8.0, 500 mm NaCl) and once with LiCl washing buffer (0.25 m LiCl, 1% NP‐40, 1% Sodium Deoxycholate, 1 mm EDTA, 10 mm Tris‐HCl pH 8.0). After washing, immunocomplexes were eluted in 120 µL of elution buffer (1% SDS, 100 mm NaHCO_3_) for 15 min at 30 °C. The immunocomplexes were reverse cross‐linked by incubating with RNase A and shaking at 65 °C overnight and then treated with proteinase K for 1 h at 60 °C. DNA was extracted with a QIAQUICK PCR Purification Kit (QIAGEN) for PCR.

### Electrophoretic Mobility Shift Assay (EMSA)

For collecting the nuclear extract, cells were incubated in the cytoplasmic buffer (10 mm Tris pH 7.5, 1.5 mm MgCl_2_, 10 mm KCl, and 0.1% Triton X‐100) for 1 min on ice. After centrifugation at 16000 g, 4 °C for 5 min, the supernatant was discarded. The pellet was washed with cytoplasmic buffer once, and then incubated with nuclear buffer (20 mm Tris pH 7.5, 20% glycerol, 1.5 mm MgCl_2_, 420 mm NaCl, 0.2 mm EDTA, and 0.1% Triton X‐100) on ice. The mixture was vortexed for 15 s every 10 min for a total of 40 min. After centrifugation for 10 min, the supernatant was collected. The FOXA1 binding site in the promoter of TGFBR1 was predicted with MoLoTool (https://molotool.autosome.org/) and the oligonucleotide sequences used were FOXA1‐TGFBR1 sense 5’‐GGTACTATTATTATTTACATTTTAGAGATGAAGA‐3’ and antisense 5’‐ TCTTCATCTCTAAAATGTAAATAATAATAGTACC‐3’. The biotinylated single‐stranded oligonucleotides were generated following the Biotin 3’ END DNA Labeling Kit (Thermo Fisher Scientific, #89818). Then, the complementary 3’ biotinylated single‐stranded oligonucleotides were annealed with the annealing buffer (10 mM Tris, 1 mM EDTA, 100 mm NaCl) in the thermocycler set to Step 1: 95 °C 5 min; Step 2: Step down 1 °C per cycle for 70 cycles; Step 3: hold at 4 °C. The same annealing protocol was used to generate the unlabeled (cold) competitor. The labeled probes were diluted to 20 fmol µL^−1^ and the unlabeled probes were diluted to 4 pmol µL^−1^ as the stock. EMSA was performed using the LightShift Chemiluminescent EMSA Kit (Thermo Fisher Scientific, #20148). Briefly, the labeling efficiency of biotinylated probes was estimated by dot blot. Binding reactions were set up in a 20 µL volume containing homemade binding buffer (12.5 mm Tris‐HCl, 62.5 mm NaCl, 1.24 mm DTT, 10% glycerol, 50 µg µL^−1^ poly dI‐dC, 0.05% Nonidet P‐40) and 10 µg of nuclear proteins with or without 1 µg FOXA1 antibodies (Abcam, ab170933). Binding reactions were pre‐incubated for 20 min at room temperature. Labeled probes were added to the binding reactions and incubated for another 20 min at room temperature. Subsequently, 5X Loading Buffer was added to the reaction and run on a 6% TBE gel using 0.5X TBE buffer (44.5 mm Tris borate, 1 mm EDTA pH 8.2 – 8.4) at 380 mA for 40 min at 4 °C at 100 V for ≈1 h. The TBE gel was pre‐run for 1 h. The DNA was transferred to a Hybond‐N+ Nylon Membrane which was pre‐soaked in 0.5X TBE for 10 min. The DNA was then UV‐crosslinked to the membrane. For detection of the biotin‐labeled DNA, the membrane was blocked for 20 min using a blocking buffer. The membrane was then incubated in conjugated/blocking buffer for 15 min. The membrane was washed four times with 1x Washing Buffer for 5 min. The membrane was then incubated in a Substrate Equilibration Buffer for 5 min and then incubated in a Substrate Working Solution for another 5 min. The biotin‐labeled DNA was visualized using the ECL.

### Proximity Ligation Assay (PLA)

PLA was conducted following the Duolink PLA Fluorescence Protocol (Sigma, DUO92008). Cells were first cultured in chamber slides (Thermo Fisher Scientific). Cells were washed once in PBS and fixed with 4% PFA in PBS for 10 min at room temperature. Afterward, cells were washed with PBS three times for 5 min. Cells were then blocked with the Duolink Blocking Solution for 60 min at 37 °C. Subsequently, the blocking buffer was removed, and the antibodies were diluted in the Duolink Antibody Diluent added to the cells, and incubated for 1 h at 37 °C. For the probe incubation, the PLUS (Sigma, DUO92002) and MINUS (Sigma, DUO92004) PLA probes were diluted in the Duolink Antibody Diluent and added to cells. The cells were incubated in a pre‐heated humidity chamber for 1 h at 37 °C. For the ligation, the PLA probe was tapped off and the Ligase in the Ligation buffer was added to the cells. The cells were incubated in a pre‐heated humidity chamber for 30 min at 37 °C. To amplify the signal, the Polymerase was diluted in the Amplification buffer and added to the cells for another 100‐min incubation in a pre‐heated humidity chamber. After final washing, the slides were mounted with DAPI (Sigma, DUO82040) and analyzed in a confocal microscope (LSM 980 with Airyscan2).

### Immunoprecipitation (IP)

Protein samples collected as above were suspended in 1 mL IP buffer (150 mm NaCl, 10 mm Tris‐Cl pH 8.0, 1 mm EDTA, 1 mm EGTA, 1% Triton X‐100, 0.5% Nonidet P‐40) with protease inhibitors and MG132. Protein samples were incubated with primary antibodies or anti‐Flag conjugated beads (Thermo Fisher Scientific, A36803) with rotation at 4 °C overnight. Afterward, the samples incubated with primary antibodies were incubated with Protein A agarose beads for another 2 h at 4 °C. The samples were washed with IP buffer with protease inhibitors once and then washed with IP buffer three times. After washing, proteins were dissolved in sample buffer (60 mm Tris‐Cl pH 6.8, 10% glycerol, 2.5% β‐mercaptoethanol, 2% SDS, 0.01% bromophenol blue) and proceeded to immunoblotting.

### Lung Morphometric Analysis

Wildtype and *Tspyl1* knockout mice at postnatal day 14 were anesthetized. The lung was collected and inflated with 4% paraformaldehyde for overnight fixation at 4 °C. The fixed lungs were dehydrated and embedded in paraffin. Sections of 5 µm thickness were cut and stained with hematoxylin and eosin (H&E) using standard procedures. The images of H&E‐stained sections were taken with Axioplan 2 imaging system (Zeiss) at 20X objective magnification and analyzed with Image J with the genotype blinded. The number of the alveoli was directly counted. To quantify the Mean Linear Intercept (MLI), equally distributed horizontal lines were placed in the image with the ‘Grid’ tool in Image J. The spacing between each line was 50 µm. The linear distance between intercepting alveolar epithelia was measured. All intercepts along the lines were measured. For each image, the mean value of the intercepts was calculated with the formula that the MLI of each image = the total length of intercepts / the number of intercepts. The MLI of each lung was calculated by adding the MLI of each image / the number of images. Eight to 12 images were taken for each mouse lung.

### Statistical Analysis

The statistical analysis of all data was performed using GraphPad Prism 8 software. Data are presented as mean ± SD and *n* is shown in figure legends. Statistical significance was determined using the two‐tailed unpaired Student's *t*‐test or two‐way ANOVA as indicated in figure legends. P < 0.05 was considered statistically significant. All cellular experiments were repeated at least once.

## Conflict of Interest

The authors declare no conflict of interest.

## Author Contributions

J.S., K.M.C., and S.Y.C. conceived the research. H.T. and S.Y.C. designed and analyzed the research. H.T., M.X.M., R.X.L, J.S., L.P. X.Z., E.H.W.L., L.Z. performed the experiment. K.M.C., M.C. advised the research and provided reagents, H.T. and S.Y.C. wrote the manuscript.

## Supporting information

Supporting Information

Supporting Table 1

## Data Availability

The data that support the findings of this study are available in the supplementary material of this article.
