# Supplementary material for: TSPYL1 as a Critical Regulator of TGFβ Signaling through Repression of TGFBR1 and TSPYL2
Source: Adv Sci (Weinh). 2024 Apr 8;11(21):2306486. doi: 10.1002/advs.202306486 (PMC11151076; doi:10.1002/advs.202306486)
Supplement: Supplementary file 1 — Supporting Information [file ADVS-11-2306486-s001.docx]

**Supporting Information**

**TSPYL1 as a Critical Regulator of TGFβ Signaling through Repression of TGFΒR1 and TSPYL2**

*Huiqi Tan, Mia Xinfang Miao, Rylee Xu Luo, Joan So, Lei Peng, Xiaoxuan Zhu, Eva Hin Wa Leung, Lina Zhu, Kui Ming Chan, Martin Cheung*, Siu Yuen Chan**

**Supporting Experimental Section**

*XTT cell proliferation assay*: After infection for 48 h, 5 × 10^3^ cells were seeded per well of a 96-well plate. The cells were subjected to XTT assay (Gold Biotechnology) following the manufacturer’s instructions.

*In vivo lung metastasis assay*: A549 cells were labelled with a firefly luciferase reporter cloned into pLVX-EF1α (Clontech) and transduced with control or TSPYL1shRNA lentiviruses. After infection, cells were trypsinized and washed with PBS twice. Cells (1.5 × 10^6^) in 200 μL PBS were injected via the tail vein for each female BALB/c nude mouse. Eight weeks later, mice were injected intraperitoneally with D-luciferin (Gold Biotechnology) and then subjected to bioluminescence imaging with PE IVIS Spectrum In Vivo Imaging System (Perkin Elmer).

*Cellular fractionation*: Cellular fractionation was carried out following previous description.^[20]^ BE(2)-C cells were digested with trypsin and washed with PBS. Cells (1 × 10^6^) were then resuspended in 200 μL Buffer A (150 mm NaCl, 50 mm HEPES pH7.5, 1 mm EDTA, 0.1% Triton X-100) supplemented with protease inhibitors on ice for 3 minutes. The lysates were centrifuged at 13000 rpm, 4 °C for 5 minutes. After centrifugation, the supernatant was collected as the detergent extractable fraction (Dt) and the insoluble pellet was washed twice with Buffer A without Triton X-100. The washed pellet was resuspended in 100 μL Buffer B (150 mm NaCl, 50 mm HEPES pH 7.5, 1 mm EDTA, 200 μg mL^-1^ RNaseA) supplemented with protease inhibitors and incubated at room temperature for another 30 minutes. The lysates were centrifuged at 14000 rpm for 5 minutes. The supernatant was collected as the RNase extractable fraction (Rn) and the pellet was collected as RNase-resistant chromatin fraction (Chr) and proceeded to immunoblotting.

**Supporting Figures**

**
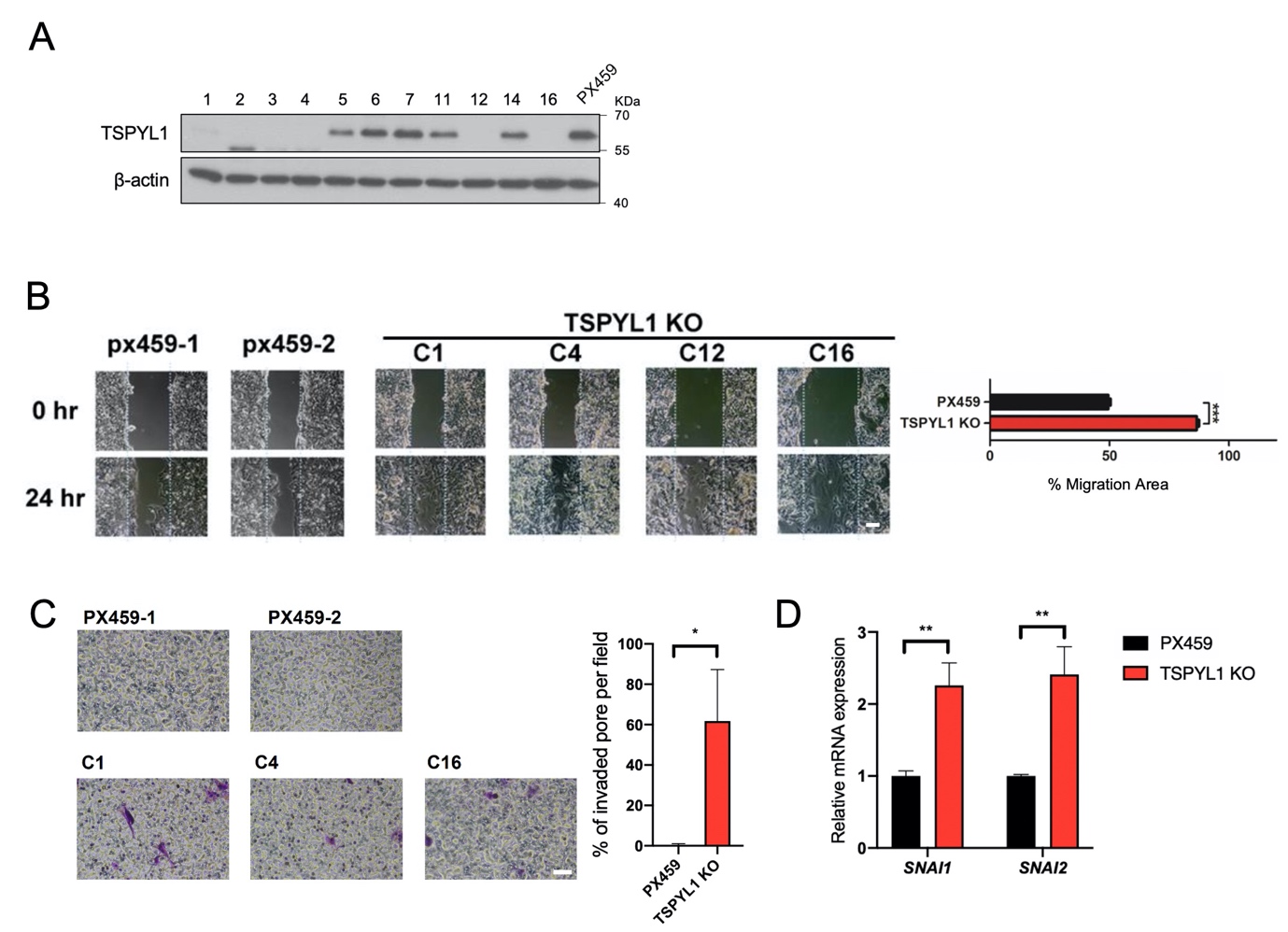
**

**Figure S1. TSPYL1 knockout drives EMT in BE(2)-C cells**

A) Screening of TSPYL1 KO BE(2)-C single clones. BE(2)-C TSPYL1 KO clones were generated by transfection with sgRNA#2 cloned into CRISPR/Cas9 vector PX459. Transfected cells were selected with puromycin and single colonies were picked. The expression of TSPYL1 was detected with immunoblot and a PX459 transfected single colony was used as the control. KO clones were verified by sequencing.

B) Confluent *TSPYL1* KO clones were treated with 10 μg mL^-1^ mitomycin C for 3 h before scratching. Images were captured immediately (0h) and 24 h afterwards. The border of migrated cells was outlined. The percentage of migrated area was measured by Image J and presented on the right. Scale bar: 100 µm.

C) Single clones of BE(2)-C were seeded onto Transwell inserts precoated with Matrigel. Transwell inserts were removed 24 h later, and cells which had migrated through the Transwell were stained with crystal violet. Images were taken under 20X objective and the number of pores with invaded cells was counted. Results are expressed as the percentage of pores counted. Data are shown as the mean ± SD, *n* = 2 PX459, 4 *TSPYL1* KO clones. *p < 0.05 by unpaired Student’s *t-*test. Scale bar: 100 µm.

D) Transcript expression of *SNAI1* and *SNAI2* in PX459 and TSPYL1 KO BE(2)-C. Data are mean ± SD, *n* = 2 PX459, 4 *TSPYL1* KO clones. **p < 0.01, by unpaired Student’s *t*-test.


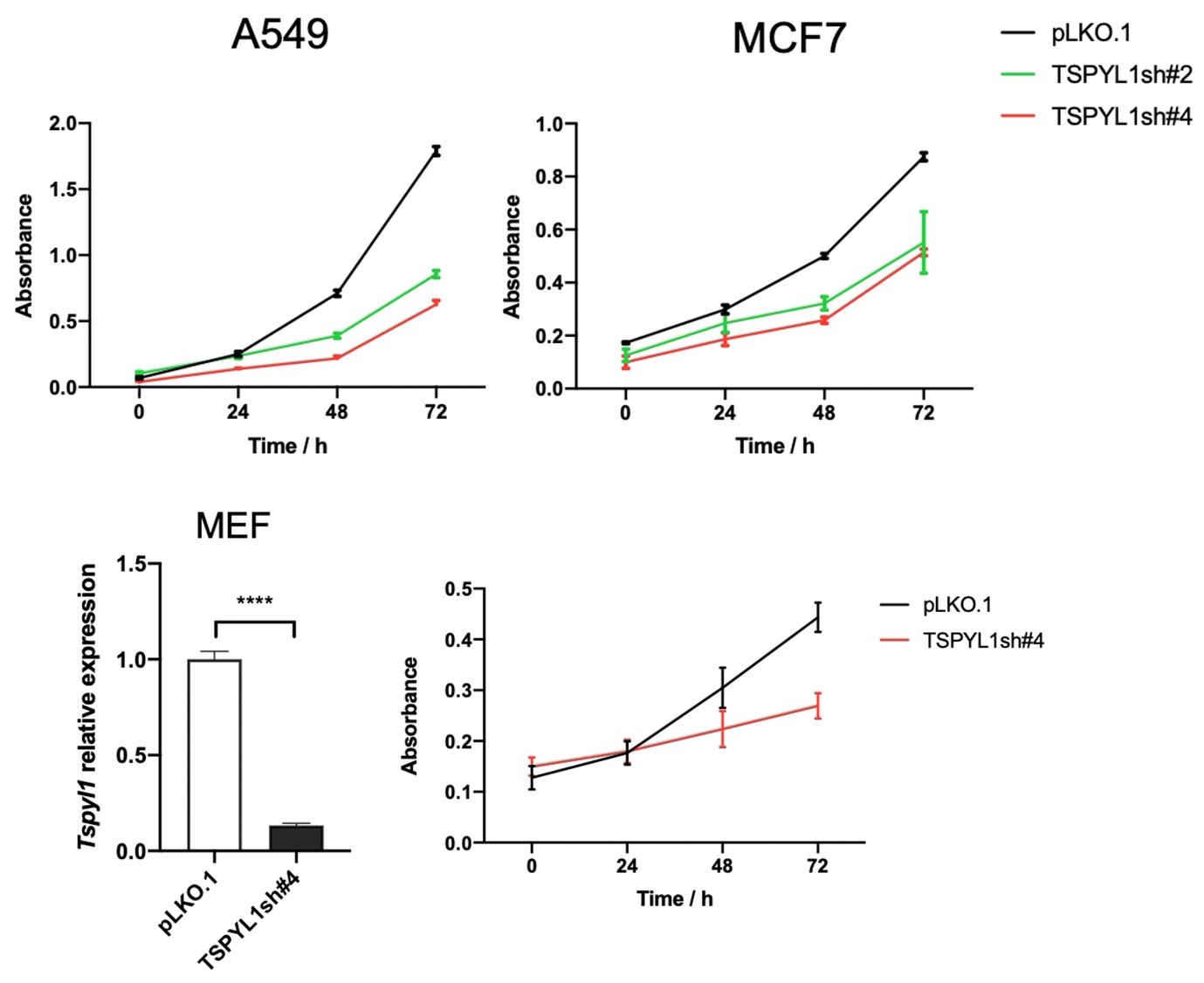


**Figure S2. TSPYL1 knockdown impairs cell proliferation**

Proliferation of control or TSPYL1 knockdown A549, MCF7 and MEF cells. Cells were transduced with control or TSPYL1shRNAs for 48 h and then subjected to XTT assay at indicated time points. *n* = 3 - 5 wells. The knockdown efficiency of *Tspyl1* in MEF was tested via qPCR. Data are mean ± SD, *n* = 4. ****p < 0.0001, by unpaired Student’s *t*-test.


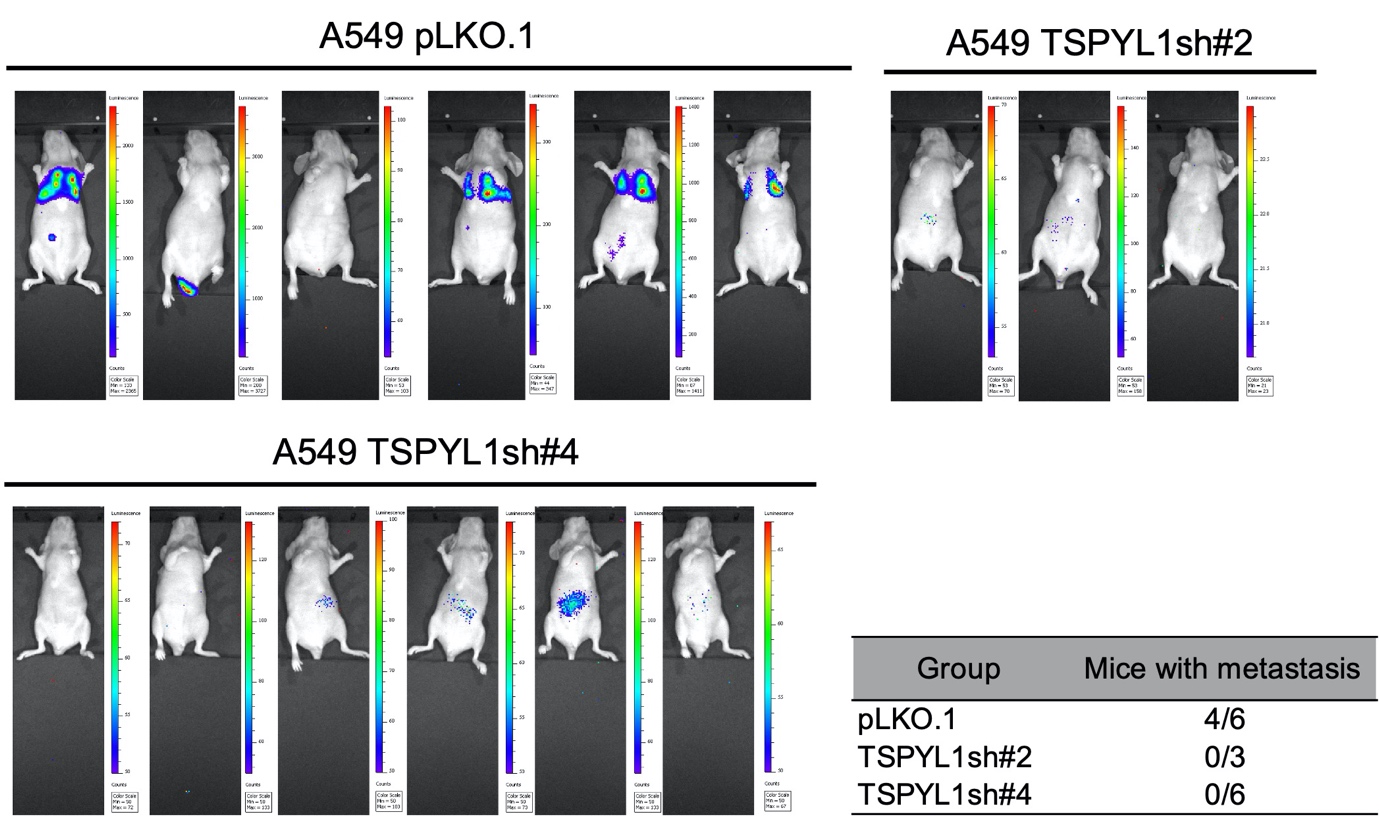


**Figure S3. TSPYL1 knockdown in A549 cells does not promote metastasis**

A549 cells were transduced with a firefly luciferase reporter together with control or TSPYL1shRNA lentiviruses. Cells were injected into BALB/c nude mice through the tail vein. Eight weeks after injection, mice were imaged by in vivo bioluminescence imaging system to check for metastasis to the lungs.

**
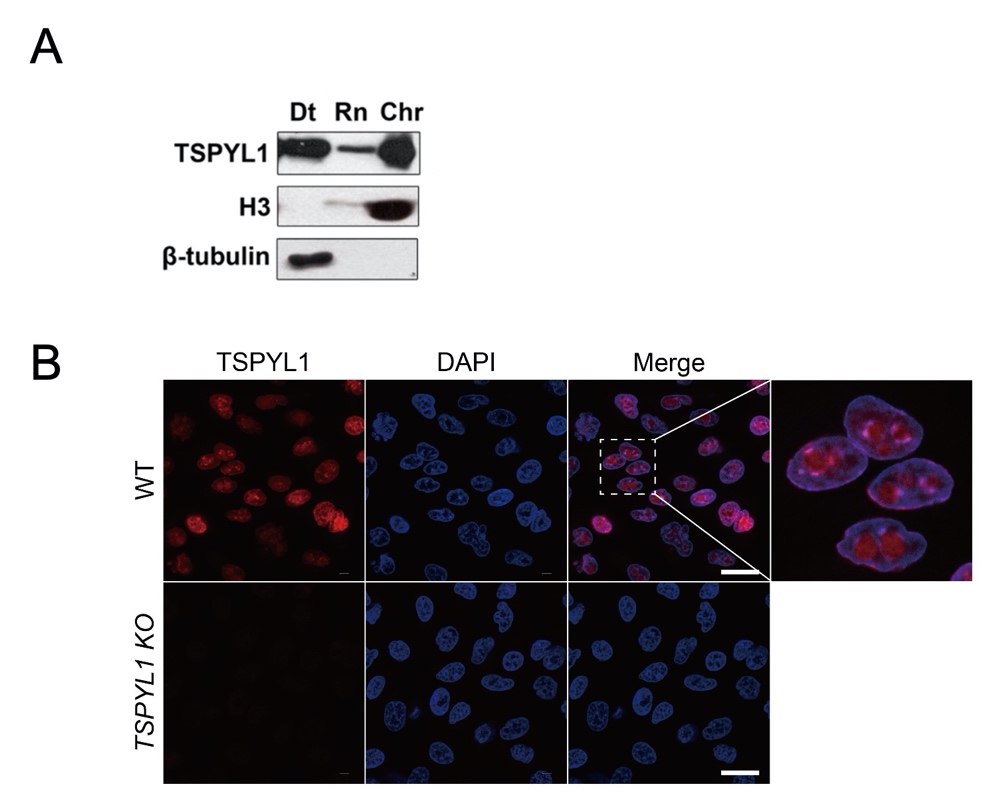
**

**Figure S4. Subcellular localization of TSPYL1**

A) Protein in different fractions was collected from BE(2)-C cells and subjected to immunoblot with indicated antibodies and shown on the right. Dt: detergent extractable fraction; Rn: RNase extractable fraction; Chr: Chromatin fraction.

B) Images of TSPYL1 immunostaining in A549 cells. Nuclei were counter-stained with DAPI. *TSPYL1* KO A549 cells generated by CRISPR/Cas9 were used as the negative control. WT, parental A549 cells. Scale bar: 20 µm.


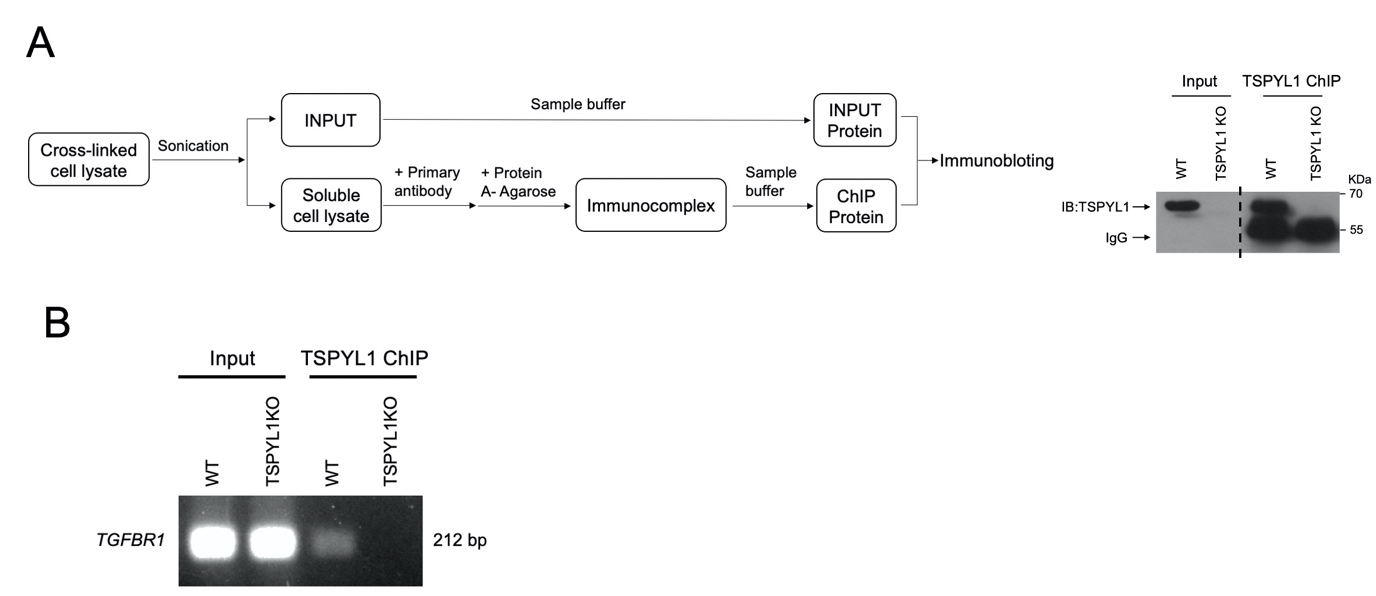


**Figure S5. Verification of TSPYL1 antibodies for cross-linking ChIP**

A) Schematic diagram of immunoprecipitation of TSPYL1 with ChIP conditions. Results of immunoblotting in A549 cells are shown on the right. *TSPYL1* KO A549 cells were used as the negative control.

B) The binding of TSPYL1 on the *TGFΒR1* promoter in A549 cells was detected by ChIP-PCR. *TSPYL1* KO A549 cells were used as the negative control.


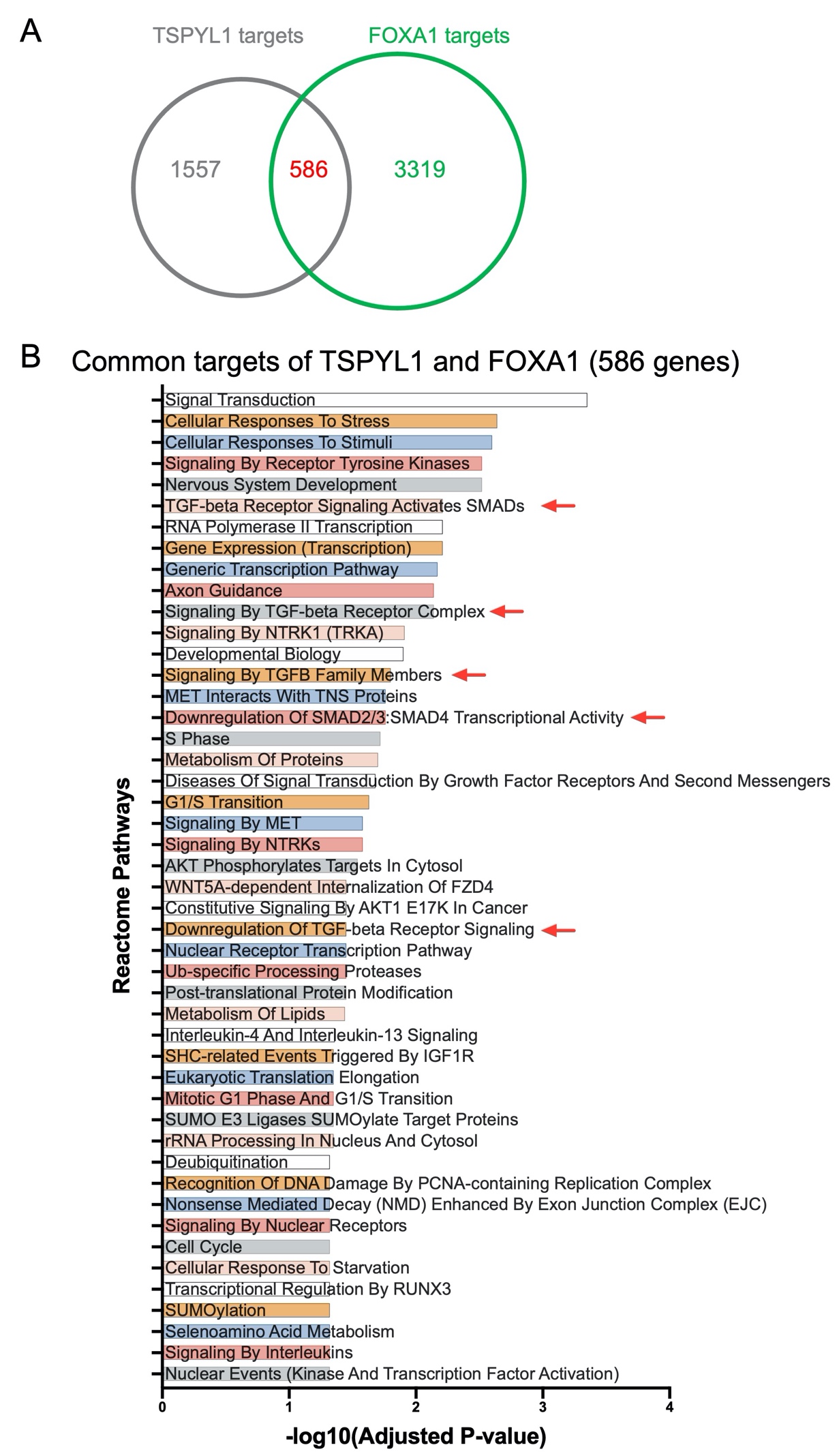


**Figure S6. Overlapping TSPYL1 and FOXA1 target genes in A549 cells**

A) Venn diagram comparing TSPYL1 and FOXA1 target genes. The FOXA1 target genes were retrieved from ChIP-atlas database (<http://chip-atlas.org/>).

B) Common target genes of TSPYL1 and FOXA1 were subjected to pathway analysis with Reactome Pathway Database (https://reactome.org/) and pathways with adjusted P-value < 0.05 were shown. Arrows indicated pathways related to TGFβ signalling.

**
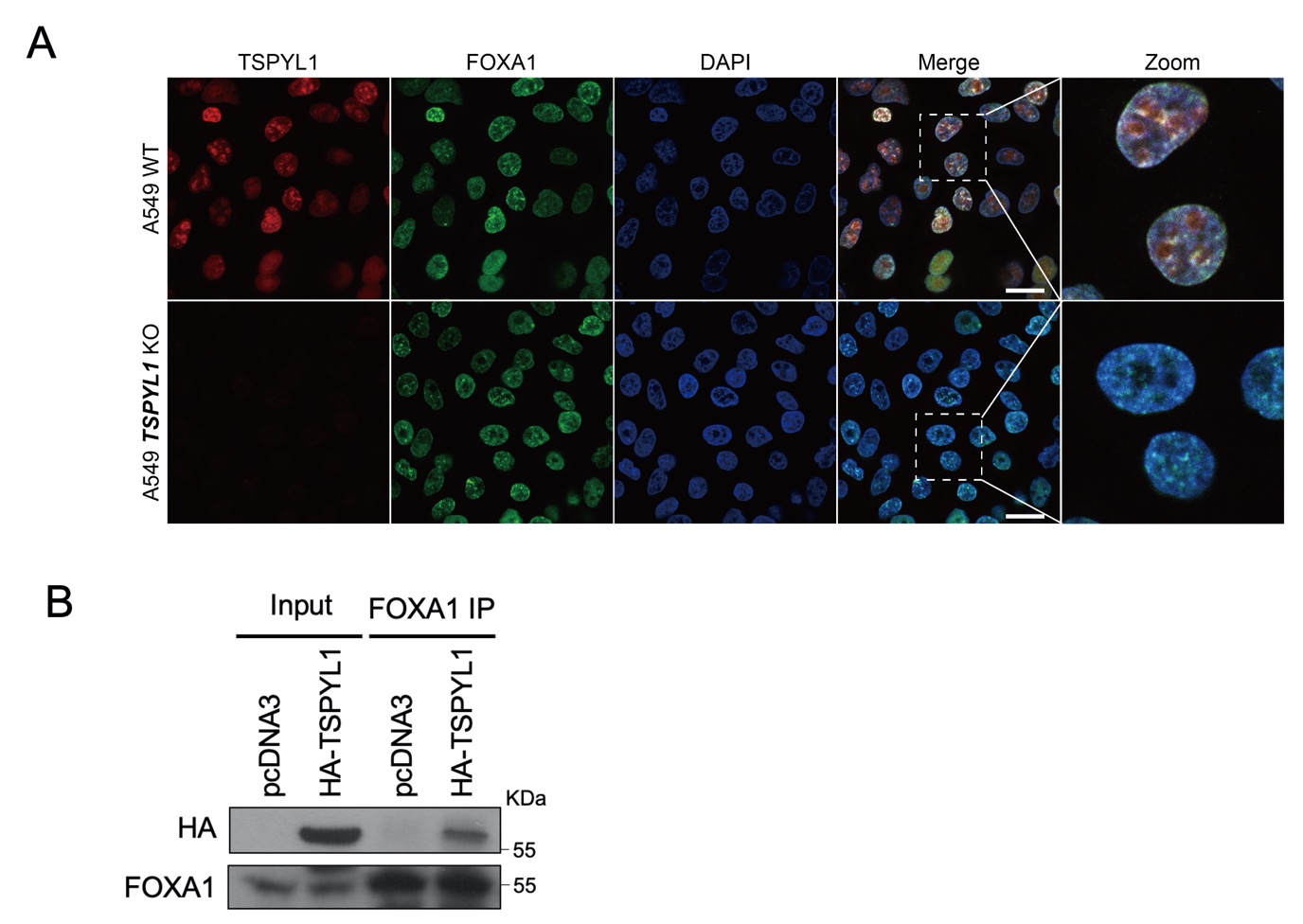
**

**Figure S7. TSPYL1 interacts with FOXA1**

A) Co-immunostaining of TSPYL1 and FOXA1 in A549. Nuclei were counterstained with DAPI. Scale bar: 20 µm.

B) Co-immunoprecipitation to show TSPYL1 interacts with FOXA1. HA-tagged full length TSPYL1 were transiently transfected into HKE293FT cells. At 72 h post-transfection, cells were subjected to immunoprecipitation (IP) with FOXA1 antibodies and immunoblotting with antibodies indicated on the left. IP complexes were electrophoresed in a 7.5% gel for better separation of FOXA1 and antibodies for IP.

**
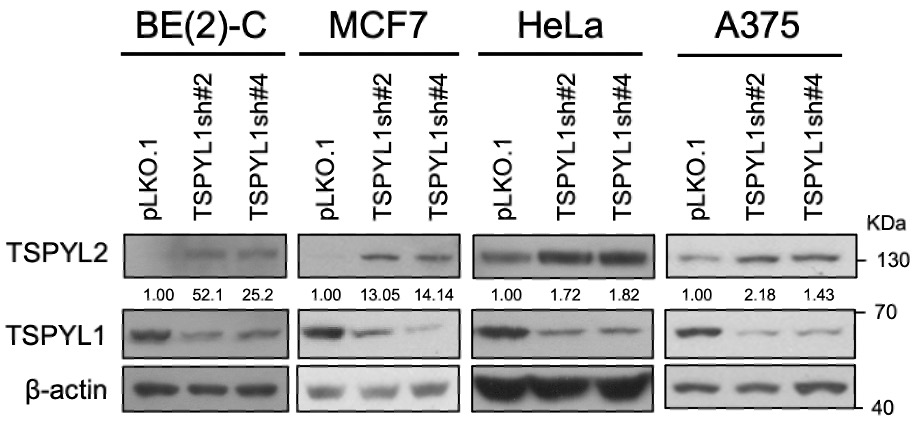
**

**Figure S8. TSPYL1 knockdown increases the protein level of TSPYL2**

Three days after transduction with indicated lentiviruses, cells as labelled on top were subjected to immunoblotting with indicated antibodies.

**Supporting Tables**

**Table S1: Target gene lists of TSPYL1 and FOXA1 in A549 cells**

(Attached as separate excel file)

**Table S2 List of cloning primers**

| **Plasmid** | **Forward primer 5’ to 3’** | **Reverse primer 5’ to 3’** |
| --- | --- | --- |
| HA-TSPYL1 | GTTAGCAACATGAGCGGCCTG | AGGAGACCTTGTGCAGGAGTA |
| HA-TSPYL1-SIDDT | GGCTGAGGCGGAGGGAGGTGAAGACAGG | CCTGCTTTCACCTCCCTCCGCCTCAGCC |
| HA-TSPYL1-ΔN | ATGCTGAATTCATGGACCCTCTGGAGGCC | AGGAGACCTTGTGCAGGAGTA |
| TSPYL1-Flag | CACACTAGTATGAGCGGCCTGGATGGGGT | CACATGCATTTACTTGTCGTCATCGTCTTTGTAGTCACCAGACTGGAACCCAA |

**Table S3. List of plasmids**

| **Plasmid** | **Sources** | **Identifier** |
| --- | --- | --- |
| pLKO.1 | Sigma | Plasmid #10878 |
| pLKO.1-TSPYL1sh#2 | Sigma | TRCN0000122627 |
| pLKO.1-TSPYL1sh#4 | Sigma | TRCN0000140845 |
| pLKO.1-TSPYL2sh#2 | This paper | N/A |
| PX459 | Gift from Dr. Feng Zhang | Plasmid #48139, [34] |
| PX459-TSPYL1sg#1 | This paper | N/A |
| PX459-TSPYL1sg#2 | This paper | N/A |
| pSIN | Gift from Dr. Qilong Ying | [35] |
| pSIN-TSPYL1-Flag | This paper | N/A |
| pBabe | Gift from Dr. | [7] |
| pBabePuro-3Xflag-Nbio-TSPYL2 | Gift from Dr. | [7] |
| pCL-Ampho | Gift from Dr. | [7] |
| psPAX2 | Addgene | Plasmid #12260 |
| pVSVG | Addgene | Plasmid #8454 |
| pcDNA3 | Invitrogen | N/A |
| pcDNA3-HA-TSPYL1-FL | This paper | N/A |
| pcDNA3-HA-TSPYL1-∆C | This paper | N/A |
| pcDNA3--HA-TSPYL1-∆N | This paper | N/A |
| pcDNA3-HA-TSPYL1-SIDDT | This paper | N/A |
| pGL3-SBE4-luc | Gift from Dr. Peter ten Diike | [16] |
| pRL-CMV | Promega | E2261 |

**Table S4 list of qPCR primers**

| **Target** | **Name of primer** | **Sequence (5' to 3')** |
| --- | --- | --- |
| *TSPYL1* | TSPYL1-q-Forward (F) | CTGGACACTGTGAATGCTCAGG |
|  | TSPYL1-q-Reverse (R) | CCCTAATCATGGCGGACAACTG |
| *CDH1* | CDH1-q-F | CAGTACAACGACCCAACCCA |
|  | CDH1-q-R | CACGCTGACCTCTAAGGTGG |
| *CDH2* | CDH1-q-F | AGCCCGGTTTCATTTGAGGG |
|  | CDH2-q-R | TTGAGGGCATTGGGATCGTC |
| *SNAI1* | SNAI1-q-F | CACTAGCCGCGCTCTTTC |
|  | SNAI1-q-R | GGTCGTAGGGCTGCTGGAA |
| *SERPINE1* | SERPINE1-q-F | ACAACAGGAGGAGAAACCCA |
|  | SERPINE1-q-R | AGCTCCTTGTACAGATGCCG |
| *TGFBR1* | TGFBR1-q-F | AGGGAAAGTCTGTCTAGCTGC |
|  | TGFBR1-q-R | GACAGAGGATCCACCGAACG |
| *SMAD2* | SMAD2-q-F | GTTCCTGCCTTTGCTGAGAC |
|  | SMAD2-q-R | TCTCTTTGCCAGGAATGCTT |
| *SMAD7* | SMAD7-q-F | TACCGTGCAGATCAGCTTTG |
|  | SMAD7-q-R | AGTTTGAAGTGTGGCCTGCT |
| *PPP2CA* | PPP2CA-q-F | GGGTCCAATGTGTGACTTGC |
|  | PPP2CA-q-R | GCAGCTTGGTTACCACAACG |
| *NOG* | NOG-q-F | GTTACAGATGTGGCTGTGGTC |
|  | NOG-q-R | TGATGGGGTACTGGATGGGAA |
| *TSPYL2* | TSPYL2-q-F | TTCCGCTACTTGACCAATCTGC |
|  | TSPYL2-q-R | GCGCTGGAACTCCTTGACAAT |
| *FOXA1* | FOXA1-q-F | GAAGATGGAAGGGCATGAAA |
|  | FOXA1-q-R | CGCTCGTAGTCATGGTGTTC |
| *Tspyl1* | Tspyl1-q-F | GCGCTGGAACTCCTTGACAAT |
|  | Tspyl1-q-R | GCGCTGGAACTCCTTGACAAT |
| *Tgfbr1* | Tgfbr1-q-F | AGGTACATGGCCCCTGAAGT |
|  | Tgfbr1-q-R | AACAGCGTCGAGCAATTTCC |
| *Smad7* | Smad7-q-F | TTGCCTCGGACAGCTCAATT |
|  | Smad7-q-R | TGCTGCGGTTGTAAACCCA |
| *HPRT* | HPRT-q-F | AACTGGAAAGAATGTCTTGATTG |
|  | HPRT-q-R | TCAAATCCAACAAAGTCTGGC |

**Table S5 List of ChIP-PCR primers**

| **Target promoter** | **Name of primer** | **Sequence (5' to 3')** |
| --- | --- | --- |
| *TGFBR1* | TGFBR1-P-F3 | CGTCATTTAAGGGGCCCAAG |
|  | TGFBR1-P-R3 | TAGACTGCCTTTTTGCTGCTC |
| *SMAD7* | SMAD7-P-F | TCCCCTAAACCACCCAGATG |
|  | SMAD7-P-R | CGCTGGTCTTCCTCTCCTTTTC |

**Table S6 List of antibodies**

| **Primary antibody** | **Source** | **Identifier** | **Usage in this study (dilution)** |
| --- | --- | --- | --- |
| Rabbit-anti-TSPYL1 | ProteinTech | Cat#13932-1-AP | IB (1:1000) |
|  |  |  | ICC (1:200) |
|  |  |  | PLA (1:200) |
|  |  |  | ChIP (1 μg for each sample) |
|  |  |  | CUT&RUN (1 μg for each sample) |
| Rabbit-anti-TSPYL2 | Bethyl Laboratories | Cat#A304-012A | IB (1:1000) |
|  |  |  | PLA (1:200) |
|  |  |  | ChIP (1 μg for each sample) |
|  |  |  | IP (1 μg for each sample) |
|  |  |  | IB (1:1000) |
| Rabbit-anti-FOXA1 | Abcam | Cat#ab170933 | ChIP (1 μg for each sample) |
|  |  |  | EMSA (1 μg for each reaction) |
|  |  |  | IP (1 μg for each reaction) |
| Mouse-anti-FOXA1 | Santa cruz | Cat#sc-514695 | IB (1:1000) |
|  |  |  | ICC (1:100) |
|  |  |  | PLA (1:100) |
| Rabbit-anti-TGFBR1 | Abcam | Cat#AB31013 | IB (1:1000) |
| Rabbit-anti-pSMAD2(Ser465/467)/SMAD3 (Ser423/425) | Cell Signalling Technology | Cat#8828 | IB (1:1000) |
| Rabbit-anti-pSMAD3(Ser423/425) | Abclonal | Cat#AP0727 | IB (1:1000) |
| Rabbit-anti-SMAD3 | Abcam | Cat#ab40854 | ChIP (1 μg for each sample) |
| Mouse-anti-SMAD4 | Santa Cruz | Cat#sc-7966 | PLA (1:100) |
| Mouse-anti-SMAD2/3 | Santa cruz | Cat#sc-133098 | IB (1:500)  PLA (1:100) |
| Mouse-anti-SMAD2 | Santa cruz | Cat#sc-393312 | IB (1:500)  PLA (1:100) |
| Mouse-anti-E-cadherin | Santa cruz | Cat#sc-8426 | IB (1:500) |
| Mouse-anti-N-cadherin | Santa cruz | Cat#sc-59987 | IB (1:500) |
| Mouse-anti-Vimentin | Santa cruz | Cat#sc-6260 | IB (1:500) |
|  |  |  | ICC (1:200) |
| Mouse-anti-ZEB1 | Santa cruz | Cat#sc-515797 | IB (1:500) |
| Goat-anti-SNAI1 | Abcam | Cat#ab53519 | IB (1:1000) |
| Rabbit-anti-HA-tag | ProteinTech | Cat#51064-2-AP | IB (1:3000) |
| Mouse-anti-Flag-tag | Sigma | Cat#P3165 | IB (1:10000) |
| Rabbit-anti-EZH2 | Cell Signalling Technology | Cat#3147 | ChIP (1 μg for each sample) |
| Rabbit-anti-H3K27me3 | Diagenode | Cat#pAb-069-050 | ChIP (1 μg for each sample) |
| Rabbit-anti-H3 | Cell Signalling Technology | Cat#4499 | IB (1:2000) |
| Mouse-anti-GAPDH | ProteinTech | Cat#60004-1-lg | IB (1:10000) |
| Mouse-anti-β-actin | ImmunoWay | Cat#YM3028 | IB (1:10000) |
| Mouse-anti-β-tubulin | Upstate | Cat#05-661 | IB (1:5000) |
| Rabbit IgG | Millipore | Cat#PP64B | ChIP (1 μg for each sample) |
|  |  |  | CUT&RUN (1 μg for each sample) |
|  |  |  | IP (1 μg for each sample) |

**Table S7 Sequencing results of *TSPYL1* knockout BE(2)-C and A549 clones**

| **Cell line** | ***TSPYL1* knockout clone** | **Sequencing result** | |
| --- | --- | --- | --- |
|  |  | **DNA level** | **Protein level** |
| BE(2)-C | C1 | c.227delG | p.Gly76AlafsX39 |
|  |  | c.223_232del | p.Gly76ValfsX36 |
|  | C4 | c.128_245del | p.Thr43ArgfsX33 |
|  |  | c.222_308del | p.Ala75TrpfsX7 |
|  | C12 | c.227_233del | p.Gly76AlafsX37 |
|  |  | c.227_236del | p.Gly76ValfsX36 |
|  | C16 | c.227delG | p.Gly76AlafsX39 |
|  |  | c.223_232del | p.Gly76ValfsX36 |
| A549 | #11 | c.80_227del | p.Asp27AlafsX39 |
|  |  | c.80_227del | p.Asp27AlafsX39 |
|  | #14 | c.68_92del | p.Gln23ProfsX4 |
|  |  | c.80_227del | p.Asp27AlafsX39 |
